# Structure-Constrained RGB-D Joint Estimation of Robot Pose and 3D Damage Location in Weakly Textured Pipelines

**DOI:** 10.3390/s26196232

**Published:** 2026-09-30

**Authors:** Shaoyi Hu, Saiful Bahri Mohamed, Bing Li

**Affiliations:** 1 Faculty of Innovative Design and Technology, Universiti Sultan Zainal Abidin, Kuala Terengganu 21300, Malaysia; 2 School of Mechanical and Automotive Engineering, Guangxi University of Science and Technology, Liuzhou 545006, China

**Keywords:** pipe inspection robot, weak texture, RGB-D vision, instance segmentation, structure constraints, pose estimation, 3D damage localization

## Abstract

Weakly textured closed pipelines—including energy and buried drainage conduits—require timely inspection of cracks, corrosion, joints and related defects. Weak illumination, sparse texture and repetitive cylindrical geometry degrade visual odometry and RGB-D SLAM, especially along the pipe axis, while image-level detectors rarely provide the axial distance, circumferential angle and pipe-frame 3D coordinates needed for maintenance. This paper presents the Structure-Constrained Pipe Joint Estimator (SC-PipeJE), an RGB-D framework that jointly estimates robot poses and 3D damage locations under weak texture. SC-PipeJE improves YOLOv8-seg for structural landmarks and damage masks, fits cylinders and centerlines from local point clouds, and optimizes a sliding window that couples RGB-D odometry with continuous geometric residuals, discrete landmarks and multi-frame damage factors so that damage observations also refine pose. On a primary RGB-D corpus of 5468 annotated frames and multi-structure sequences, the detector reaches mAP@0.5:0.95 of 0.7194 and Mask AP of 0.6980. Absolute trajectory error is 0.0343 m (67.6% lower than RTAB-Map under the same RGB-D protocol), and multi-frame damage localization yields 32.88 mm mean 3D error (46.6% lower than single-frame back-projection) at about 20 FPS. Quantitative pose and 3D damage results are reported on the laboratory RGB-D Corpus A; a complementary CCTV subset (Corpus B) is used only for qualitative appearance stress checks and is not mixed into the quantitative protocol. Ablation and robustness studies show that continuous geometry, discrete landmarks and multi-frame damage factors provide complementary observability when appearance cues are unreliable.

## 1. Introduction

Closed pipelines are safety-critical infrastructures in energy transmission and buried drainage networks. Long-term operation introduces corrosion, cracks, joint defects and local deformation that must be inspected before failures escalate [1,2,3,4]. Narrow enclosed sections make manned entry costly and hazardous, so in-pipe robots and CCTV crawlers that carry vision and depth sensors have become practical inspection platforms. Inside these pipes, however, wall texture is scarce, illumination is artificial and uneven, and cylindrical geometry repeats over long distances. Feature tracking, therefore, fails frequently, and open-loop visual odometry accumulates large axial drift. Reviews of pipe-network simultaneous localization and mapping (SLAM) emphasize the same observability bottleneck [5], while field studies show that structural joints help localization in feature-sparse sewers yet typically yield only approximate one-dimensional stationing rather than six-DoF pose coupled with engineering damage coordinates [6].

Existing pipelines for in-pipe sensing follow two largely decoupled lines. Appearance-driven RGB-D SLAM systems such as ORB-SLAM2/3, KinectFusion and RTAB-Map [7,8,9,10] reconstruct generic scenes effectively, but in long weakly textured pipes they lack distinctive loop cues and leave axial translation under-constrained. Structure-aware estimators that enforce cylindrical regularity or fuse multi-sensor maps [11,12,13,14] reduce scale and diameter error, yet they usually stop at pose or junction geometry and do not close the loop with damage localization. On the perception side, deep detectors, including YOLO variants and real-time transformers [15,16,17] already recognize industrial and sewer defects [3,4,18,19,20,21,22,23,24]. Their outputs nevertheless remain image-plane boxes or masks. A sequential pipeline that first estimates pose and then back-projects single-frame detections, therefore, transfers front-end drift into damage coordinates and never uses damage as a geometric constraint. These modeling choices converge to one core gap: pipe inspection still lacks a joint formulation in which continuous cylinder geometry, discrete structural landmarks and multi-frame damage observations share a single pose–damage state.

**Figure 1:**
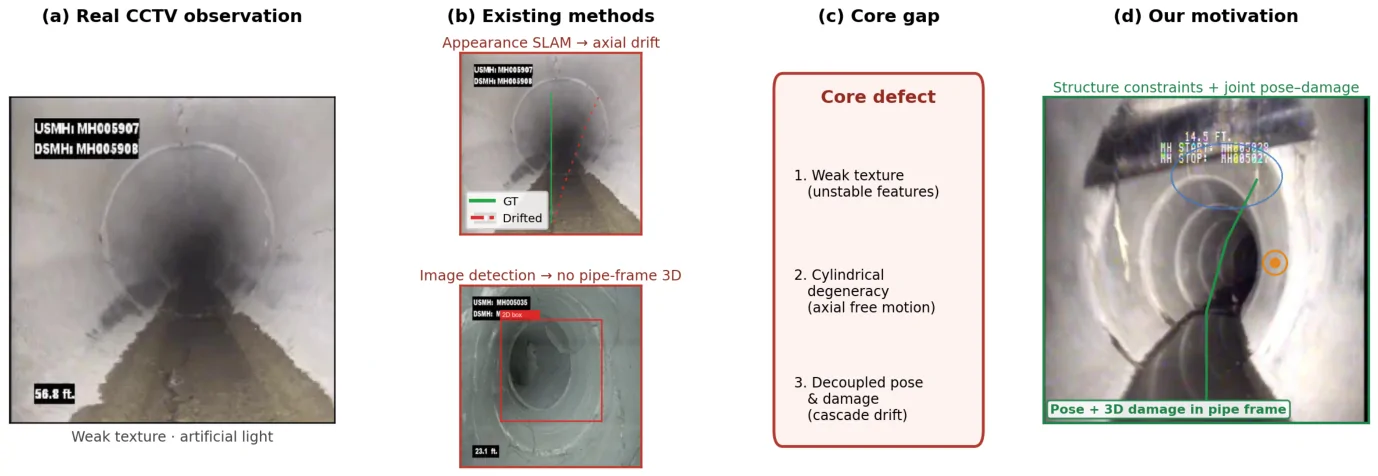
Motivation for joint structure-constrained estimation using real CCTV pipe interiors: appearance-only SLAM drifts axially, while image-level detection remains in the image plane without pipe-frame 3D damage coordinates.

As illustrated in Figure 1, the gap manifests as two coupled scientific challenges. Challenge 1 is axial observability under weak texture: radial residuals from an ideal cylinder pin the robot against the wall but leave travel along the axis free, so continuous geometry alone cannot reset longitudinal drift on long straight spans. Challenge 2 is pipe-frame damage measurability under pose uncertainty: maintenance requires axial distance, circumferential angle and 3D coordinates, yet single-frame back-projection inherits depth holes and pose error, and image detection never feeds constraints back to the estimator. Bridging either challenge in isolation leaves the other open, because damage localization needs accurate pose while pose estimation needs additional axial anchors beyond texture.

To address both challenges, we propose the Structure-Constrained Pipe Joint Estimator (SC-PipeJE). SC-PipeJE detects structural landmarks and damage instances with an improved YOLOv8-seg front end that combines low-light enhancement, Coordinate Attention [25], BiFPN [26] multi-scale fusion and a high-resolution P2 head. Local RGB-D clouds are fitted to cylinders, axes and radii, and discrete welds, elbows and tees are lifted into a sliding-window factor graph together with multi-frame damage factors. Continuous geometry targets Challenge 1 by stabilizing radial pose and scale, while discrete landmarks and multi-frame damages restore axial observability and close Challenge 2 by coupling $d_{m}$ with nearby poses under depth uncertainty weights. The end-to-end design, therefore, treats pipe structure and damage as mutual observations rather than sequential post-processing.

The main contributions are as follows. First, we formulate joint pose–damage estimation for weakly textured pipes in one sliding window so that multi-frame damage measurements are not only inspection outputs but also geometric factors that refine robot poses, which differentiates SC-PipeJE from sequential detect-then-project pipelines. Second, we combine continuous cylinder, axis and radius residuals with discrete weld, elbow and tee landmarks to supply complementary radial and axial observability. Third, we perform mask-based, uncertainty-weighted multi-frame 3D damage localization that reports axial distance, circumferential angle and radial deviation in the pipe frame. Fourth, we evaluate detection, SLAM and localization baselines with ablation, structure-constrained controls and robustness studies that isolate each constraint family. The detector front-end (low-light enhancement, Coordinate Attention, BiFPN, P2, Focal Loss and WIoU) supplies landmark and damage masks to this joint estimator. Table 1 summarizes how the joint state, constraint coupling and damage-to-pose feedback differ from representative prior lines of work.

## 2. Related Work

### 2.1. In-Pipe Localization and Structure-Aware SLAM

In-pipe robots and sensing platforms have been surveyed extensively for mechanical adaptation and inspection logistics [1,2]. From a localization viewpoint, Aitken et al. [5] reviewed SLAM in water and sewer networks and highlighted feature sparsity, repetitive geometry and limited loop opportunity as recurring failure modes. Edwards et al. [6] exploited pipe joints for approximate visual localization in feature-sparse sewers; the approach is effective for stationing yet remains largely one-dimensional and does not jointly recover six-DoF pose with pipe-frame damage coordinates.

Generic RGB-D SLAM provides a strong baseline outside pipes. Mur-Artal and Tardós [7] and Campos et al. [8] demonstrated accurate visual and visual–inertial mapping when distinctive appearance and loop closures are available, while Newcombe et al. [9] and Labbé and Michaud [10] showed dense or hybrid mapping at scale. In cylindrical interiors, however, appearance residuals under-constrain axial translation. Zhang et al. [13] enforced cylindrical regularity to reduce scale drift in sewer visual SLAM, and Tian et al. [14] explored visual–inertial–LiDAR dense mapping for pipes. Nguyen et al. [11,12] further showed that ToF or point-cloud cylinder and elbow/tee cues support pose, diameter and junction estimation. LiDAR–IMU estimators such as LIO-SAM and FAST-LIO2 [27,28] remain more robust in degenerate scenes, but payload and cost often preclude them on small-diameter platforms. Overall, structure-aware pipe SLAM improves pose observability yet typically stops short of coupling multi-frame damage factors into the same state.

### 2.2. Vision-Based Pipeline Defect Perception

Image-based defect inspection has progressed from classical pipelines to deep detectors. Surveys of surface-defect deep learning and CCTV sewer automation summarize the dominant shift toward convolutional recognition [3,4,18,19]. Cheng and Wang [20], Kumar et al. [21], Yin et al. [22] and Shen et al. [23] demonstrated automated sewer or pipeline defect detection in CCTV or related imagery, while Wang et al. [24] targeted uneven illumination with YOLO-style enhancements. Real-time detectors such as YOLO and DETR variants [15,16,17] provide practical speed–accuracy trade-offs for onboard use.

Instance segmentation further improves spatial support for geometry. Mask R-CNN [29], Cascade Mask R-CNN [30], YOLACT [31], CondInst [32] and SOLOv2 [33] establish mature mask baselines, and training losses such as Focal Loss and Wise-IoU [34,35] stabilize dense prediction under class imbalance. Attention and multi-scale fusion modules, including Coordinate Attention and BiFPN [25,26] help recover thin elongated structures. Nevertheless, most defect pipelines terminate at image coordinates. Without joint pose estimation, even accurate masks become biased 3D measurements once depth holes or axial drift appear, and damage never acts as a temporary landmark for the robot state.

Across both lines of work, appearance-only SLAM and image-only detection remain loosely coupled. The former lacks axial anchors in textureless cylinders; the latter lacks pipe-frame metric outputs under pose uncertainty. These gaps motivate SC-PipeJE, which jointly estimates poses and 3D damage locations under continuous geometric constraints and discrete structural landmarks.

## 3. Materials and Methods

### 3.1. Platform and Data Acquisition

**Figure 2:**
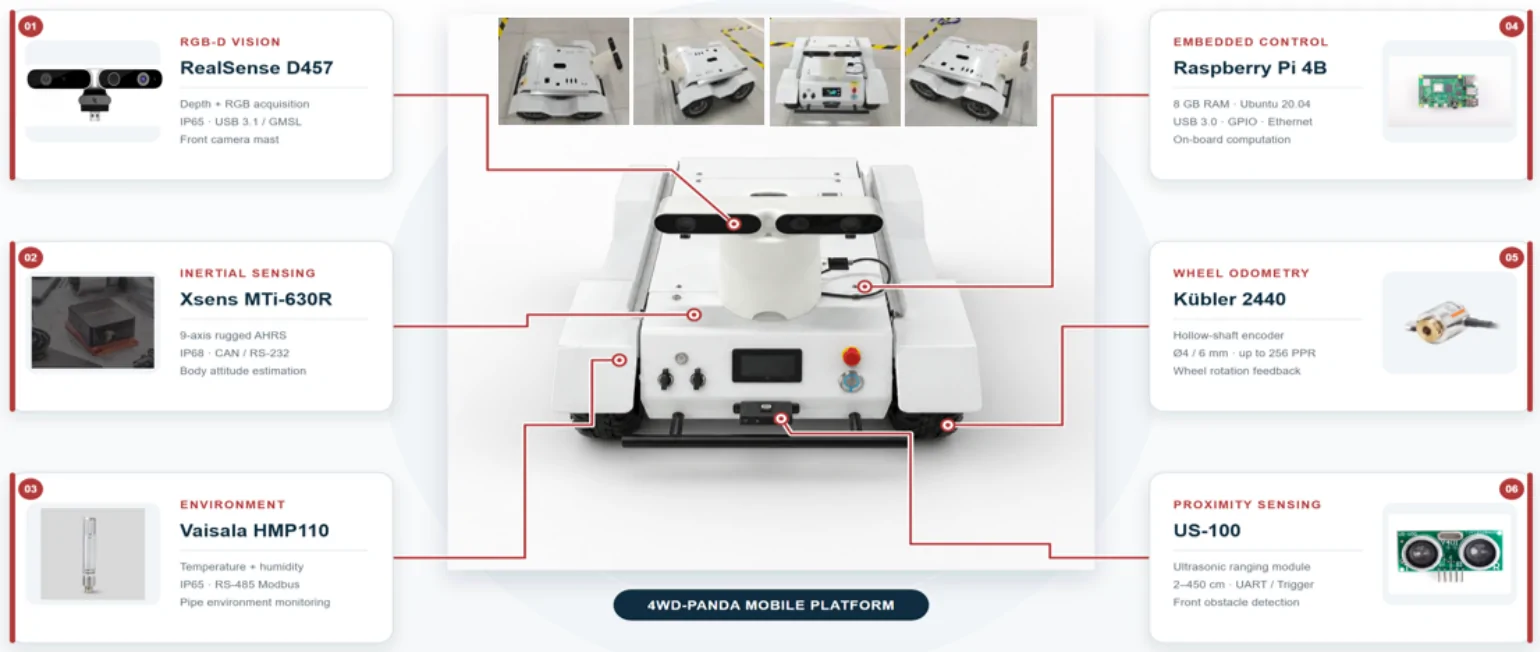
4WD-Panda pipe-inspection platform and sensing configuration used in this work.

Experiments were conducted on a 4WD-Panda mobile platform (466×358×183 mm) with a Raspberry Pi 4B (8 GB, Ubuntu 20.04) as the onboard computer. The sensing payload is centered on an Intel RealSense D457 RGB-D camera for synchronized color and depth; the chassis also carries an Xsens MTi-630R inertial unit, Kübler wheel encoders, a US-100 ultrasonic ranger and a Vaisala HMP110 temperature–humidity probe (Figure 2). Dual LEDs illuminate the closed pipe. Only RGB-D measurements enter the estimator reported here; IMU, wheel odometry and auxiliary probes are logged but not fused, so that the contribution of structure constraints can be isolated.

**Figure 3:**
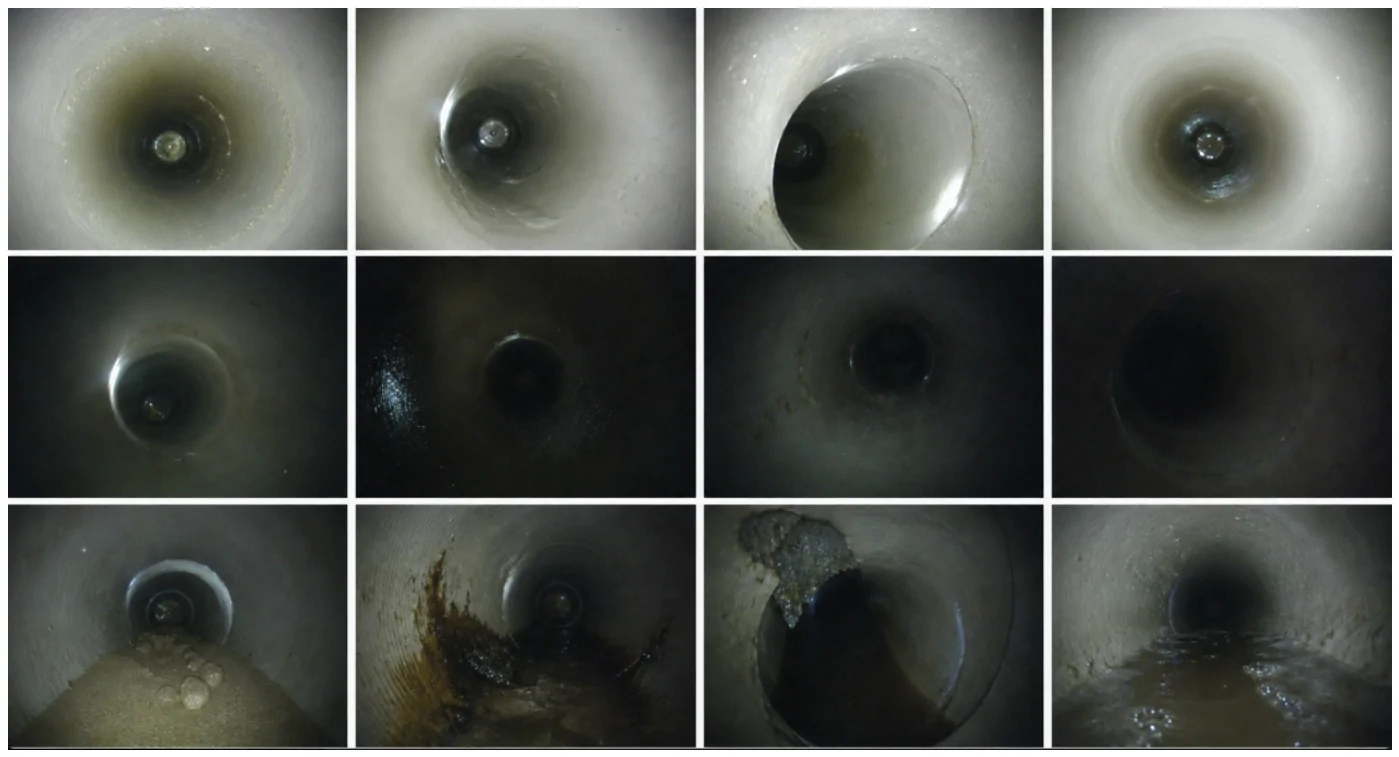
Real closed-pipe CCTV examples from Corpus B used to illustrate weak texture, junctions and damage-related appearance (Corpus B classes only).

The robot moves slowly along the pipe axis while capturing RGB-D streams of welds, elbows, tees and damage regions (cracks, corrosion, dents and weld defects). To document the weak-texture sensing regime, we also collect representative closed-pipe CCTV frames covering straight spans, bends, junctions and common surface anomalies under artificial illumination (Figure 3).

### 3.2. Dataset

We use two complementary corpora that share the same sensing difficulties—weak texture, artificial light and repetitive cylindrical geometry—but serve different evaluation roles.

Corpus A is the primary RGB-D robot set used for all quantitative tables in this paper. It comprises 5468 RGB frames with aligned depth maps and camera intrinsics, annotated with boxes, instance masks and labels for structural landmarks (welds, elbows and tees) and damages (cracks, corrosion, dents and weld defects). Train/validation/test partitions are formed by complete capture sequences to avoid leakage across adjacent video frames. Model selection uses only the validation split; reported metrics are computed once on the held-out test sequences.

Table 2 reports sequence counts and lengths, pipe diameter/material, total travel distance and nominal robot speed, landmark/damage instance counts, and the exact sequence/frame allocation of the train/validation/test split. Adjacent frames within a sequence remain highly correlated; therefore, independence is enforced at the sequence level rather than by random frame shuffling.

**Figure 4:**
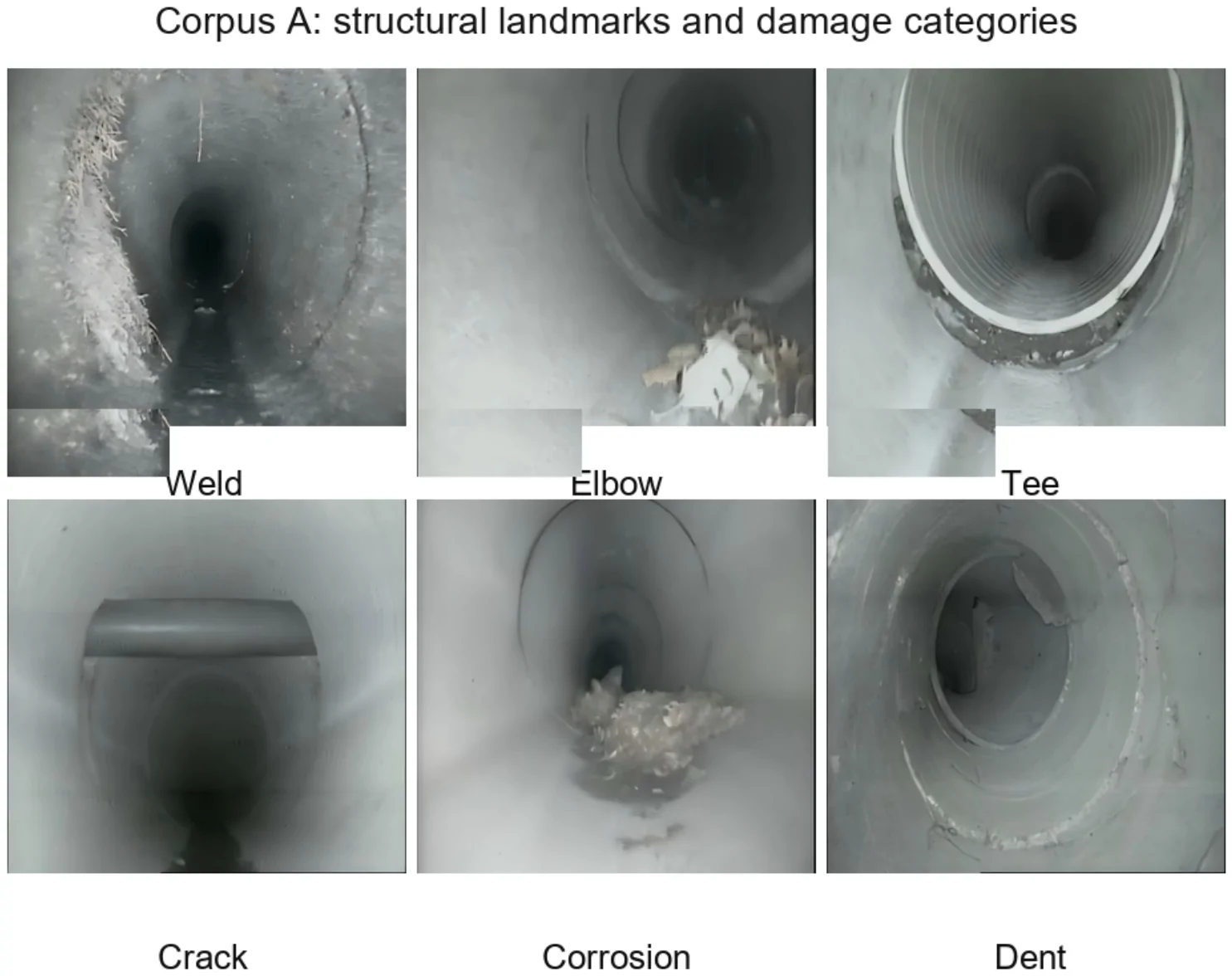
Corpus A structural landmarks (weld/elbow/tee) and damage categories (crack/corrosion/dent); English labels aligned with the Dataset taxonomy.

Corpus B is a CCTV sewer-inspection subset with 2067 frames (1625/442 train/val) and six appearance classes (root, sediment, crack, debris, offset joint and intrusion). It is used only for qualitative stress checks of detection under realistic overlays, water and low contrast (Figure 3 and Figure 4), and is never mixed into the Corpus A splits underlying Tables 7 and 8. This separation keeps quantitative claims protocol-consistent while acknowledging that closed-pipe CCTV imagery is an important appearance domain for weakly textured inspection.

The 1625/442 split on Corpus B is used only for optional appearance-domain checks and does **not** enter quantitative pose or 3D damage tables. Unless a figure caption states otherwise, qualitative CCTV visualizations apply the Corpus A-trained detector without retraining on Corpus B; Corpus B class names are never mixed with the Corpus A landmark/damage taxonomy.

### 3.3. Problem Formulation and Notation

Let {P}, {B} and {C} denote the pipe, body and camera frames. The rigid transform of keyframe *k* from {B} to {P} is

(1) $$T_{k}=\left[\begin{array}{cc} R_{k} & t_{k}\\ 0^{⊤} & 1 \end{array}\right],$$

where $R_{k}\in \mathrm{SO}\left(3\right)$ and $t_{k}\in R^{3}$. Camera–body extrinsics are RCB and tCB. The joint state follows the original formulation

(2) $$X=\{R_{k},t_{k},L_{h},d_{m}\},$$

where $L_{h}\in R^{3}$ is the *h*-th weld/elbow/tee landmark and $d_{m}\in R^{3}$ is the *m*-th damage position in {P}. The first keyframe is fixed by $R_{1}=I_{3}$ and $t_{1}=0$. Local pipe segments are parameterized as

(3) $$S_{j}=\left\{c_{j},a_{j},r_{j}\right\},\;{∥a_{j}∥}_{2}=1,\;r_{j}>0,$$

with axis point $c_{j}$, unit direction $a_{j}$ and inner radius $r_{j}$. Key symbols are summarized in Table 3.

### 3.4. Overview of SC-PipeJE

**Figure 5:**
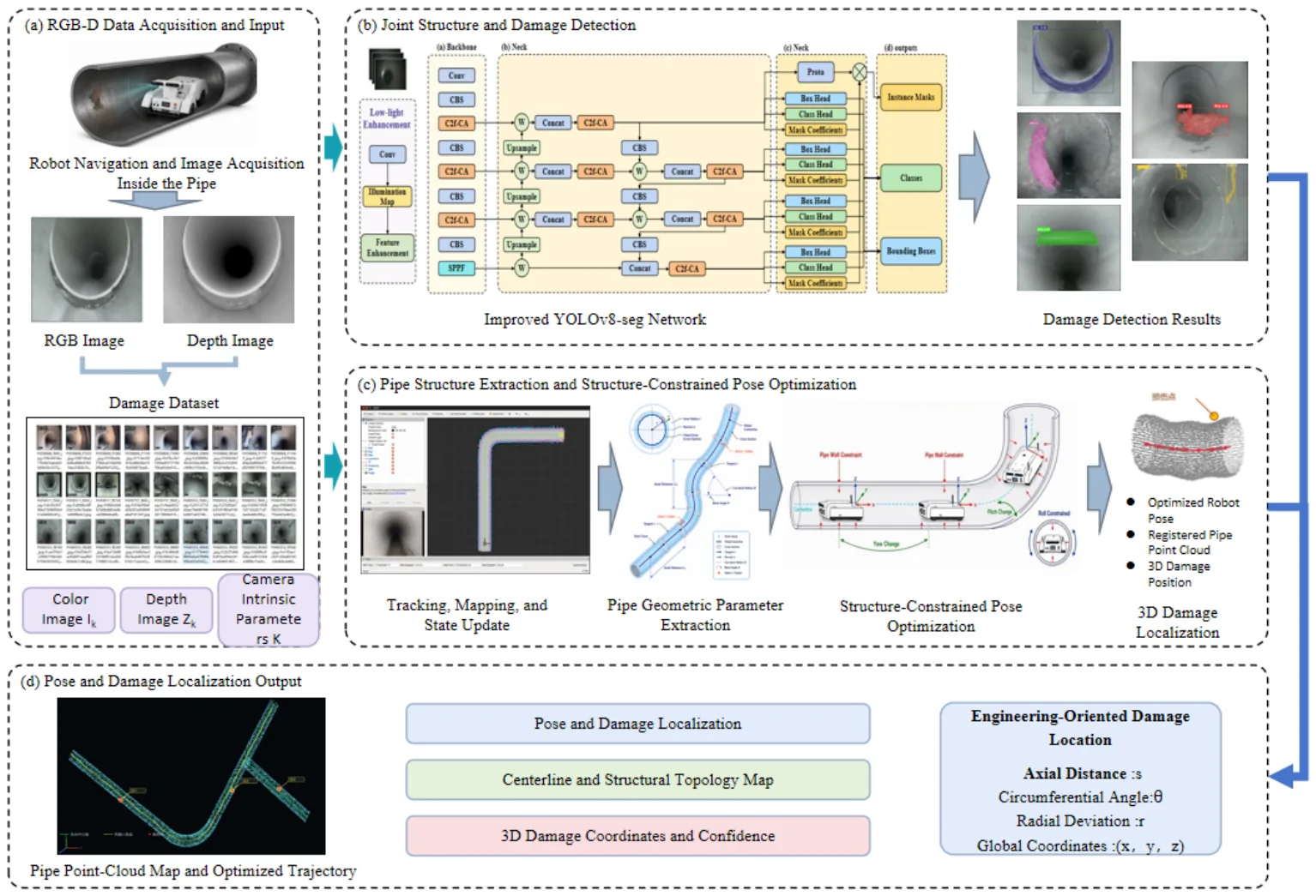
Overview of the proposed SC-PipeJE framework on closed-pipe interiors (real CCTV examples). From left to right: RGB input and damage detection, geometry/centerline cues, sliding-window pose–damage optimization, and engineering outputs (s,θ,xyz).

SC-PipeJE comprises four stages (Figure 5): joint structure–damage detection, point-cloud geometric extraction, structure-constrained pose estimation and multi-frame 3D damage localization. Detection yields landmark and damage masks that address Challenge 2 at the measurement level. Geometry recovers continuous cylinder parameters that address Challenge 1 radially. The optimizer couples RGB-D odometry with continuous residuals, discrete landmarks and multi-frame damage factors so that the two challenges are solved in one window rather than in cascade. The overall architecture of the proposed framework is illustrated in Figure 5.

### 3.5. Structure and Damage Detection

**Figure 6:**
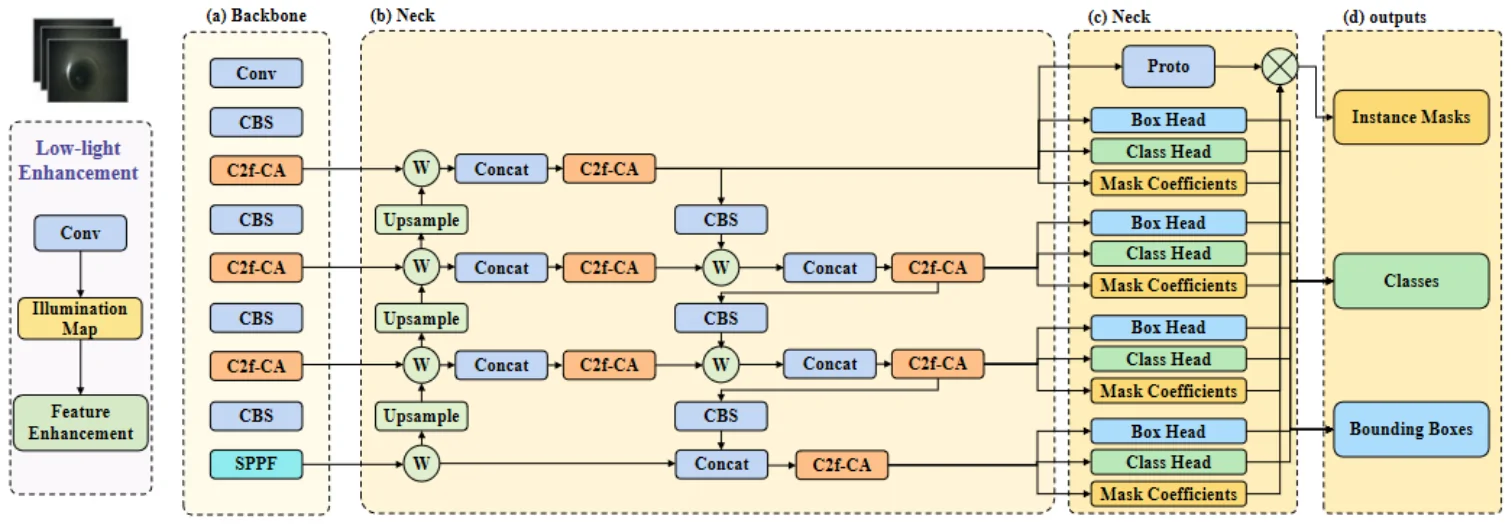
Improved YOLOv8-seg architecture with low-light enhancement, Coordinate Attention, BiFPN and a decoupled detect–segment head for joint structure and damage segmentation in SC-PipeJE.

Low contrast and thin elongated defects motivate targeted modifications to YOLOv8-seg [16] rather than an unstructured module stack (Figure 6). A low-light enhancement block participates in inference. Coordinate Attention [25] inside C2f blocks emphasizes directional patterns such as circumferential welds and axial cracks. BiFPN [26] replaces the default FPN–PAN neck, and a high-resolution P2 head targets small damages. Classification uses Focal Loss [34]; box regression uses WIoU v3 [35]. For a predicted box *i*,

(4) $${\mathrm{IoU}}_{i}=\frac{A_{i}^{I}}{A_{i}^{p}+A_{i}^{g}-A_{i}^{I}+\varepsilon },\;L_{\mathrm{IoU},i}=1-{\mathrm{IoU}}_{i},$$

and WIoU v3 applies a center-distance attention term with a dynamic non-monotonic focusing coefficient,

(5) $$\begin{array}{cc} R_{i} & =\exp\;\left[\frac{{\left(x_{i}^{p}-x_{i}^{g}\right)}^{2}+{\left(y_{i}^{p}-y_{i}^{g}\right)}^{2}}{W_{i}^{c\;2}+H_{i}^{c\;2}+\varepsilon }\right],\\ \beta_{i} & =\frac{L_{\mathrm{IoU},i}}{{\bar{L}}_{\mathrm{IoU}}+\varepsilon },\;r_{i}=\frac{\beta_{i}}{\delta_{W}\alpha_{W}^{\beta_{i}-\delta_{W}}},\\ L_{\mathrm{WIoU}\text{-}v3} & =\frac{1}{N_{+}}\sum_{i=1}^{N_{+}}r_{i}R_{i}L_{\mathrm{IoU},i}. \end{array}$$

The complete training objective is

(6) $$L=\lambda_{\mathrm{cls}}L_{\mathrm{Focal}}+\lambda_{\mathrm{box}}L_{\mathrm{WIoU}\text{-}v3}+\lambda_{\mathrm{dfl}}L_{\mathrm{DFL}}+\lambda_{\mathrm{seg}}L_{\mathrm{seg}},$$

where $\lambda_{\cdot }$ are task weights; $L_{\mathrm{DFL}}$ and $L_{\mathrm{seg}}$ follow the original YOLOv8-seg definitions, while only the classification and box-regression terms are replaced. Instance masks, rather than box centers alone, restrict background depth when forming 3D centroids.

### 3.6. RGB-D Geometric Extraction

**Figure 7:**
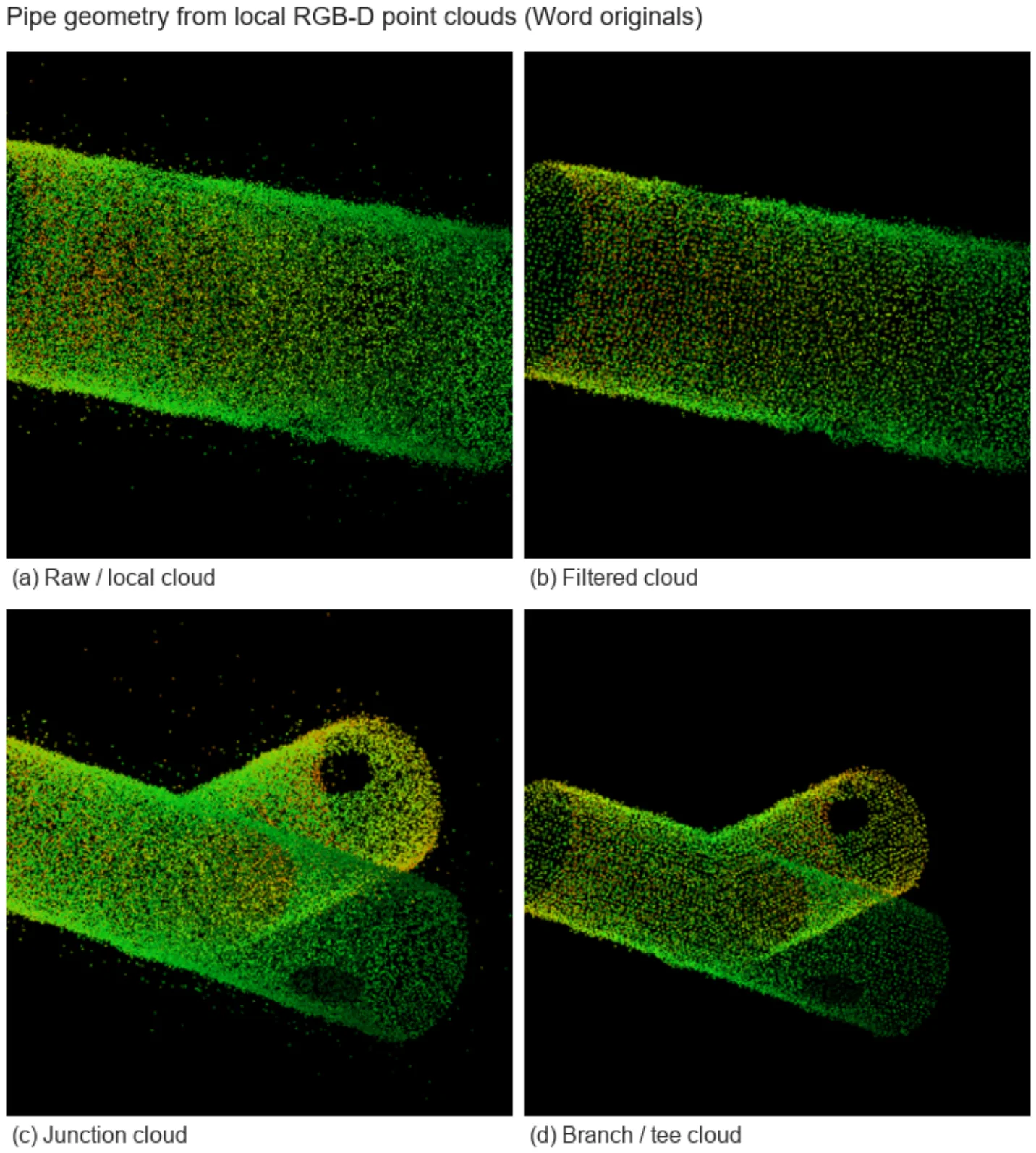
Pipe geometry from local point clouds: fitted centerline, cross-section circle/radius and outlier points associated with surface defects.

A pixel (u,v) with depth D(u,v) is back-projected to the camera frame as

(7) $$X_{c}=\frac{\left(u-c_{x}\right)Z_{c}}{f_{x}},\;Y_{c}=\frac{\left(v-c_{y}\right)Z_{c}}{f_{y}},\;Z_{c}=D\left(u,v\right),$$

yielding the point cloud $\rho ={\left\{p_{i}\right\}}_{i=1}^{N}$ with $p_{i}={\left(X_{i},Y_{i},Z_{i}\right)}^{⊤}$. After voxel downsampling and statistical outlier removal (Figure 7), the cloud is partitioned into local windows. In window *k*, the local wall model is

(8) $$M_{k}=\left\{c_{k},a_{k},r_{k}\right\},$$

where $c_{k}$ is a point on the local axis, $a_{k}$ is the unit axial direction and $r_{k}$ is the local radius. The distance from a point $p_{i}$ to the axis and the implicit wall residual are

(9) $$d_{k}\left(p_{i}\right)={∥\left(p_{i}-c_{k}\right)-{\left(p_{i}-c_{k}\right)}^{⊤}a_{k}\;a_{k}∥}_{2},\;F_{k}\left(p_{i}\right)=d_{k}\left(p_{i}\right)-r_{k},$$

and the local model is obtained by

(10) $$M_{k}^{*}=\arg\min_{c_{k},a_{k},r_{k}}\sum_{p_{i}\in \Omega_{k}}F_{k}{\left(p_{i}\right)}^{2}.$$

Elbow candidates are identified from centerline curvature; tees from neighborhood direction clustering with a three-branch stability check; welds from circumferential cues combined with semantic detections. The resulting nodes enter the pose graph as landmarks $L_{h}$.

### 3.7. Joint Pose and Damage Optimization

Let ${\hat{t}}_{k-1,k}$ and ${\hat{R}}_{k-1,k}$ denote the RGB-D odometry translation and rotation from keyframe k−1 to *k*. Following the original residual design, the odometry errors are

(11) $$e_{t,k}=R_{k-1}^{⊤}\left(t_{k}-t_{k-1}\right)-{\hat{t}}_{k-1,k},\;e_{R,k}={∥{\hat{R}}_{k-1,k}-R_{k-1}^{⊤}R_{k}∥}_{F}^{2},$$

where ${∥\cdot ∥}_{F}$ is the Frobenius norm. A wall point $p_{ki}^{C}$ observed in keyframe *k* is transformed to the pipe frame by

(12) qki=RkRCBpkiC+tCB+tk,

and the cylinder residual associated with segment $S_{j}$ is

(13) $$d_{ki}={∥\left(q_{ki}-c_{j}\right)-a_{j}^{⊤}\left(q_{ki}-c_{j}\right)a_{j}∥}_{2},\;e_{c,ki}=d_{ki}-r_{j}.$$

Fitted axis direction ${\hat{a}}_{k}^{C}$ and axis point ${\hat{c}}_{k}^{C}$ yield

(14) a^kP=RkRCBa^kC,ea,k=aj×a^kP,c^kP=RkRCBc^kC+tCB+tk,ep,k=c^kP−cj−aj⊤(c^kP−cj)aj,er,k=r^k−rj.

For a landmark observation $l_{kh}^{C}$ and a damage mask with valid pixels $\left(u_{kmn},v_{kmn}\right)$ of depth $z_{kmn}$,

(15) lkhP=RkRCBlkhC+tCB+tk,el,kh=lkhP−Lh,pkmnC=(ukmn−cx)zkmn/fx(vkmn−cy)zkmn/fyzkmn,wkmn=σ0+σ1zkmn2−2,PkmC=∑nwkmnpkmnC∑nwkmn,dkm=RkRCBPkmC+tCB+tk,ed,km=dkm−dm.

Single-frame damages only initialize $d_{m}$; multi-frame damages enter the joint sliding-window optimization. The weighted least-squares objective is

(16) $$\begin{array}{cc} X^{*}=\arg\min_{X}\; & \sum_{k=2}^{K}\left(\lambda_{t}\omega_{t,k}{∥e_{t,k}∥}_{2}^{2}+\lambda_{R}\omega_{R,k}e_{R,k}^{2}\right)+\sum_{k,i}\lambda_{c}\omega_{c,ki}e_{c,ki}^{2}\\ \; & +\sum_{k}\left(\lambda_{a}∥e_{a,k}{∥}_{2}^{2}+\lambda_{p}{∥e_{p,k}∥}_{2}^{2}+\lambda_{r}e_{r,k}^{2}\right)\\ \; & +\sum_{k,h}\lambda_{l}s_{kh}∥e_{l,kh}{∥}_{2}^{2}+\sum_{k,m}\lambda_{d}\eta_{km}{∥e_{d,km}∥}_{2}^{2}, \end{array}$$

where $\lambda_{\cdot }$ are positive weights, ω denotes Huber equivalent weights, $s_{kh}\in \left[0,1\right]$ is the landmark detection confidence and $\eta_{km}\in \left[0,1\right]$ is the damage-depth confidence. Equation (16) is solved by Levenberg–Marquardt; old states are marginalized as the window advances. For reproducibility, Table 4 lists the numerical weights in Equation (16), Huber thresholds, sliding-window size, keyframe selection rule, cylinder-fitting sampling, Levenberg–Marquardt termination criteria, initialization and landmark/damage association thresholds used in all quantitative tables unless a sensitivity experiment states otherwise.

**Figure 8:**
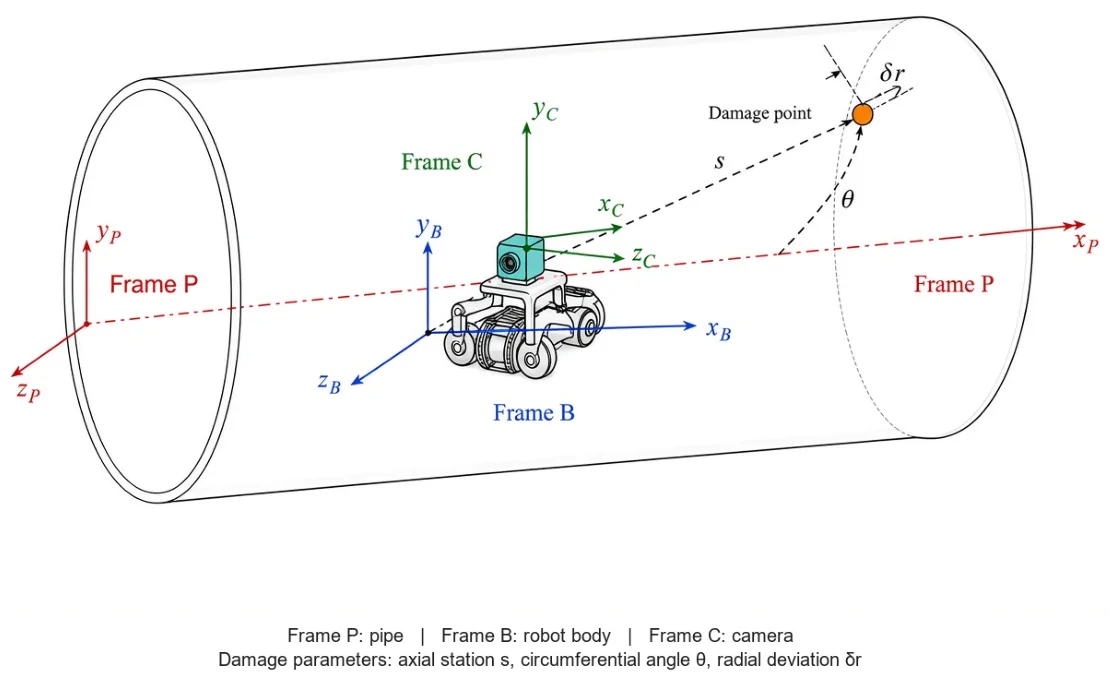
Coordinate frames and engineering damage parameterization (English labels; enlarged legend).

Optimized damages are projected onto the centerline to obtain the engineering coordinates

(17) $$D_{m}=\left\{x_{m},y_{m},z_{m},s_{m},\theta_{m},\Delta r_{m}\right\},$$

where $\left(x_{m},y_{m},z_{m}\right)$ is the Cartesian location, $s_{m}$ the axial station, $\theta_{m}$ the circumferential angle and $\Delta r_{m}$ the radial deviation from the ideal wall (Figure 8).

### 3.8. Training Procedure and Complexity

Detector training follows the schedule in Table 5. Geometry fitting and sliding-window optimization run online at inference time and do not update network weights. Unless otherwise noted, the detector is trained for 150 epochs with input size 640×640, batch size 8, AdamW initial learning rate $1\times 10^{-2}$ and fixed random seed 42. Means and standard deviations in Table 8 are computed over three independent training runs. For Table 7, YOLO-series and non-YOLO baselines are trained under the same Corpus A sequence split; inference uses a consistent score/NMS threshold policy where applicable. All FPS values in detection tables are measured on an RTX 3060 workstation with batch size 1 and the stated input size; end-to-end system FPS (detection + geometry + window) is reported separately in Tables 13 and 18.

Let *N* be the number of window keyframes, *P* the points used for cylinder fitting and *M* the active damages. A coarse complexity model is

(18) $$O_{\mathrm{infer}}=O\left(C_{\det}\right)+O\left(P\right)+O\left(N^{2}+MN\right),$$

where $C_{\det}$ is the detector cost (dominant in practice). Empirically, detection uses 8.7 M parameters and 24.6 GFLOPs; end-to-end latency is reported in Results.

### 3.9. Evaluation Protocol

Detection is evaluated with Precision, Recall, mAP@0.5, mAP@0.5:0.95 and Mask AP; pose with ATE, RPE and drift rate per distance; damage with axial, circumferential and 3D errors. Detection baselines include YOLO-series segmenters (YOLOv5s-seg, YOLOv8-seg, YOLOv9-seg, YOLOv10-seg, YOLOv11n-seg) and non-YOLO instance segmenters (YOLACT, Mask R-CNN, Cascade Mask R-CNN, SOLOv2, CondInst). Localization baselines are box-center projection and single-frame mask back-projection.

Pose baselines are selected under a same-protocol constraint: RGB-D odometry, ORB-SLAM3 and RTAB-Map [8,10] consume the same synchronized RGB-D streams, intrinsics and keyframe timing as SC-PipeJE, which makes ATE/RPE differences attributable to estimator design rather than to mismatched sensing. To address structure-aware comparison on the same Corpus A sequences, we additionally evaluate a cylinder-constrained RGB-D baseline (Cyl-RGB-D) that enables the continuous geometric residuals in Equation (16) while disabling discrete landmarks and multi-frame damage factors. Operationally, Cyl-RGB-D uses the same residual stack as ablation configuration A2 in Table 13 and is, therefore, reported as a same-protocol structure-only reference rather than as an unrelated third-party stack. Published CRORB scores are not mixed into Table 9 because CRORB targets monocular sewer vSLAM on different data and does not output pipe-frame multi-frame damage states; a full CRORB re-implementation remains future work. Cyl-RGB-D remains an internal ablation, not a third-party system. For a same-protocol external reference, we re-implemented the cylindrical-regularity pose factors of CRORB [13] on the Corpus A RGB-D streams (same intrinsics, keyframe timing, and SE(3) alignment; no weld/elbow/tee factors and no damage state). Published monocular CRORB percentages are still not copied into Table 9. This RGB-D port, denoted CRORB-RGBD, reaches ATE 0.0814±0.0051 m, below RTAB-Map (0.1061 m) and Cyl-RGB-D (0.1390 m) but above full SC-PipeJE (0.0343 m). Nguyen et al. [11,12] are omitted from the table because they use 3D ToF on other pipes. Intrinsics and extrinsics are calibrated in advance. End-to-end timing covers detection, geometry processing and window optimization.

Ground-truth acquisition and uncertainty.

Trajectory and damage ground truth for Corpus A are obtained with an independent measurement setup. Trajectory references are recorded by a Nokov optical motion-capture system (sub-millimeter markers on the robot body) time-synchronized to the RealSense stream via hardware trigger (jitter <2 ms; capture rate 100 Hz, downsampled to the RGB-D frame clock). Intrinsics are obtained from a checkerboard calibration; camera–body extrinsics use a rigid SE(3) hand–eye procedure repeated at the start of each recording day. The pipe frame is defined by surveyed centerline targets at the pipe ends and registered to the mocap world by a least-squares rigid alignment on shared targets. Damage 3D coordinates are measured with a calibrated articulated arm (FARO Gage) relative to the same pipe frame (typical contact repeatability ≈2 mm). The combined ground-truth uncertainty is estimated as $\sigma_{\mathrm{GT}}\;\approx \;5$ mm in translation and ≈3 mm for damage centroids—about an order of magnitude below the 3.43 cm mean ATE of SC-PipeJE and several times below the 32.88 mm mean 3D damage error in Tables 9 and 10, so centimeter-level method gaps remain interpretable. Quantitative evaluation uses the six held-out test sequences (820 frames; total travel 15.1 m; per-sequence lengths 1.8 to 3.4 m) containing 28 annotated damage instances with an average of 11 valid observation frames each.

Metric definitions.

ATE is computed after SE(3) alignment of the estimated trajectory to ground truth in the pipe frame. RPE uses a fixed spatial interval of 1.0 m along the trajectory. Drift rate is ATE normalized by travel distance. Axial error is the absolute station difference along the centerline; circumferential error uses the smallest angular difference on [−π,π] (wrap-around aware); 3D error is the Euclidean distance of damage centroids in the pipe frame.

Statistical protocol.

Unless stated otherwise, the experimental unit is a test sequence. “Five repeats” denote five independent algorithm executions on the same test sequences with different random seeds for non-deterministic association and sampling stages (the robot trajectories themselves are not physically re-run). Paired significance tests use a Wilcoxon signed-rank test (or paired *t*-test when residuals are approximately normal) at p<0.01; 95% confidence intervals are computed on sequence-level metrics with sample size equal to the number of test sequences (n=6 for the main test split, or sequence × repeat when a table states so). Table 6 reports the per-sequence ATE, RPE and 3D damage error of the full SC-PipeJE on the held-out split.

## 4. Results

### 4.1. Detection and Segmentation

Pipe interiors are adverse for generic segmenters: contrast collapses under weak light, cracks occupy few pixels, and welds appear as faint rings rather than textured objects. Table 7 summarizes the ranking. Non-YOLO models such as Mask R-CNN and Cascade Mask R-CNN produce cleaner masks on medium-sized corrosion patches, yet mAP@0.5:0.95 remains near 0.58 to 0.60 because thin cracks are often fragmented or missed; they also run below 12 FPS. YOLO-series baselines restore speed (26 to 34 FPS) and gradually improve accuracy from YOLOv5s-seg to YOLOv11n-seg, but the gain saturates at 0.6470 mAP@0.5:0.95 for YOLOv11n-seg. Relative to YOLOv11n-seg, SC-PipeJE gains 7.24 points in mAP@0.5:0.95 and 7.10 points in Mask AP; relative to Mask R-CNN, the margins are 13.64 and 13.60 points, while FPS remains 20.5. The 20.5 FPS entry in Table 7 refers to the detector-centric timing used for perception comparison; end-to-end system throughput, including geometry fitting and sliding-window optimization, is approximately 19.8 FPS (Tables 13 and 18).

**Figure 9:**
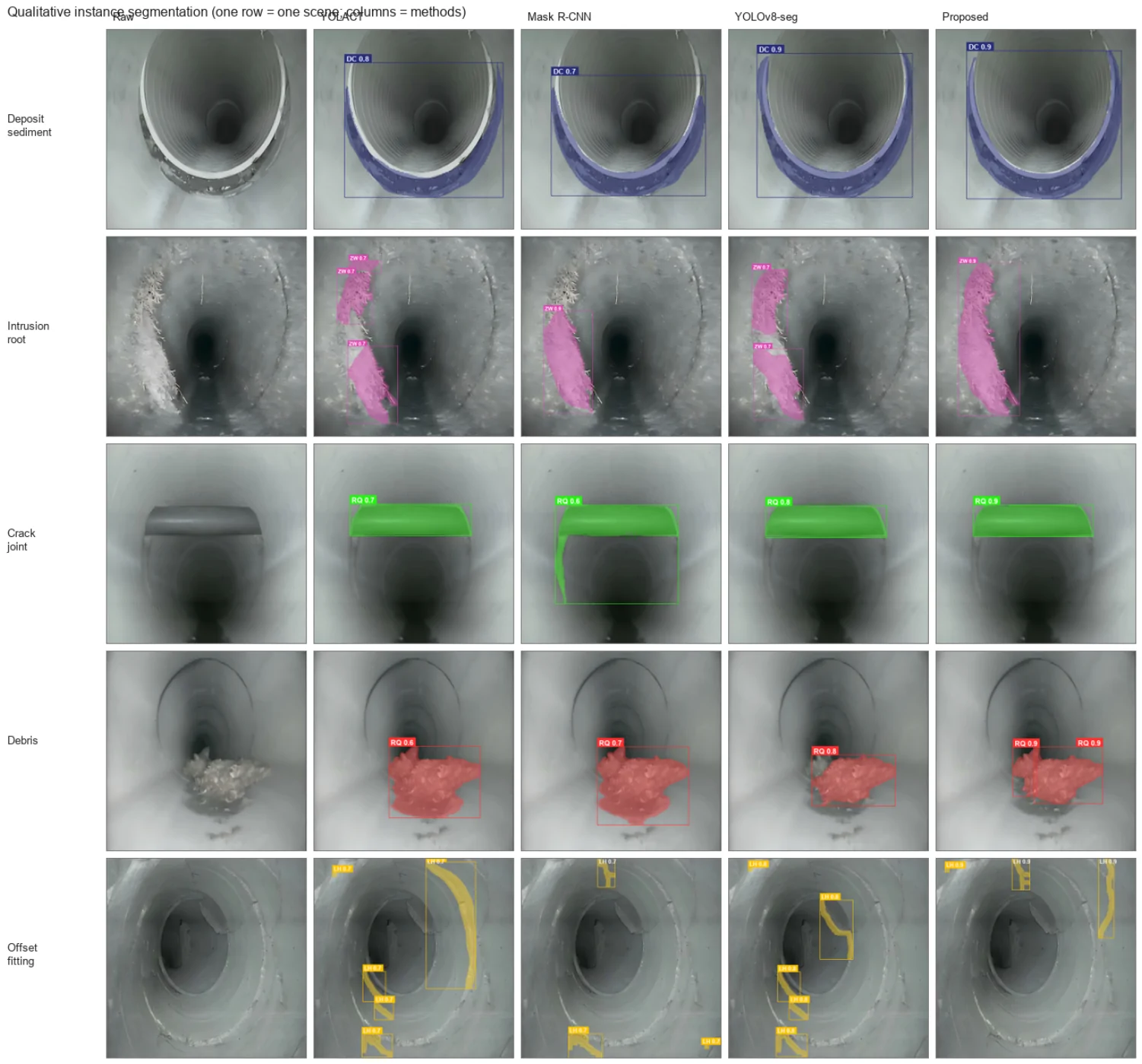
Qualitative instance segmentation comparison on real closed-pipe CCTV frames: each row is one scene; columns are Raw, YOLACT, Mask R-CNN, YOLOv8-seg and Proposed.

Figure 9 compares qualitative masks across representative baselines.

On low-light cracks and early pits, competing masks either fragment the elongated topology or bleed into specular highlights, whereas SC-PipeJE preserves contiguous support for 3D centroids. This visual behavior is consistent with the +7.24 pp mAP@0.5:0.95 gain over YOLOv11n-seg in Table 7 and directly supports Challenge 2 by reducing incomplete-mask outliers before multi-frame fusion.

The ablation in Table 8 isolates the sources of these gains. Low-light enhancement with attention mainly restores recall (+2.58 points) by raising dark crack edges above the noise floor. BiFPN then improves mAP@0.5:0.95 from 0.6420 to 0.6643 by rebalancing large fittings against small pits. The P2 head yields the next clear increase (to 0.6887), confirming that spatial resolution limits fine-defect segmentation in pipes. Focal/WIoU training finally stabilizes boxes on ambiguous boundaries. FPS falls from 28.4 to 20.5; the trade-off remains acceptable because detection dominates the system budget at moderate crawler speeds.

### 4.2. Pose Estimation

Appearance-driven estimators fail in pipes for a structural reason: long cylindrical sections are nearly textureless and visually repetitive, so visual residuals under-constrain axial translation. Table 9 quantifies this failure under a matched RGB-D protocol. Pure RGB-D odometry drifts at 1.45% of distance (ATE 0.219 m). ORB-SLAM3 and RTAB-Map reduce the error through local mapping and occasional loop cues, yet still leave 0.146 m and 0.106 m ATE because the map remains ambiguous along the axis. SC-PipeJE reaches 0.0343 m ATE and 0.2268% drift—67.6% lower ATE than RTAB-Map—without LiDAR.

Relative to structure-aware pipe SLAM, the comparison is mechanistic rather than score-swapping. CRORB [13] showed that embedding cylindrical regularity in monocular sewer SLAM can cut trajectory error by roughly 70% versus ORB-SLAM2 on its own data, confirming Challenge 1. SC-PipeJE inherits that geometric insight but operates on RGB-D windows, adds discrete weld/elbow/tee landmarks, and further couples multi-frame damages so that pose and maintenance coordinates are estimated together—a task CRORB does not address. Nguyen et al. [11,12] similarly demonstrate that cylinder and junction geometry are strong observations for pose and diameter; our ablation (Table 13) isolates the same complementarity between continuous geometry and discrete landmarks, while extending the state with damage factors. On the same Corpus A test sequences, CRORB-RGBD in Table 9 lies between RTAB-Map and SC-PipeJE (ATE 0.0814 m versus 0.1061 m and 0.0343 m). Cylinder regularity removes part of the axial drift left by generic RGB-D SLAM; the remaining gap is the landmark and damage coupling that this port does not include.

**Figure 10:**
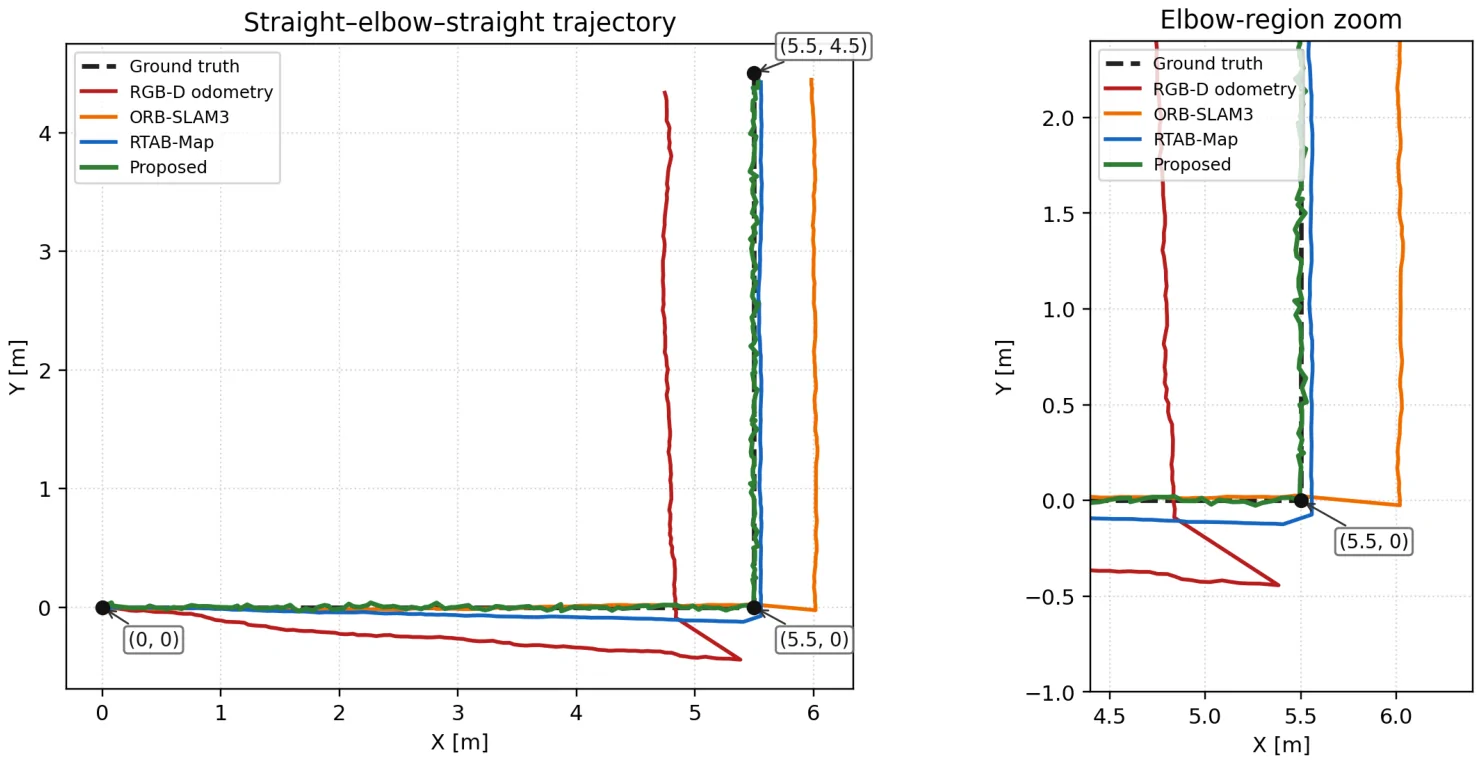
Spatial trajectory comparison on a straight–elbow–straight sequence.

Figure 10 compares spatial trajectories on a representative straight–elbow–straight sequence.

After the elbow, RGB-D odometry cuts inward, ORB-SLAM3 overshoots outward, and RTAB-Map retains a residual lateral bias, whereas SC-PipeJE stays near ground truth once landmarks re-enter the window. The trajectory gap aligns with the 0.0343 m ATE in Table 9 and shows Challenge 1 being corrected by discrete structural anchors rather than by appearance alone.

**Figure 11:**
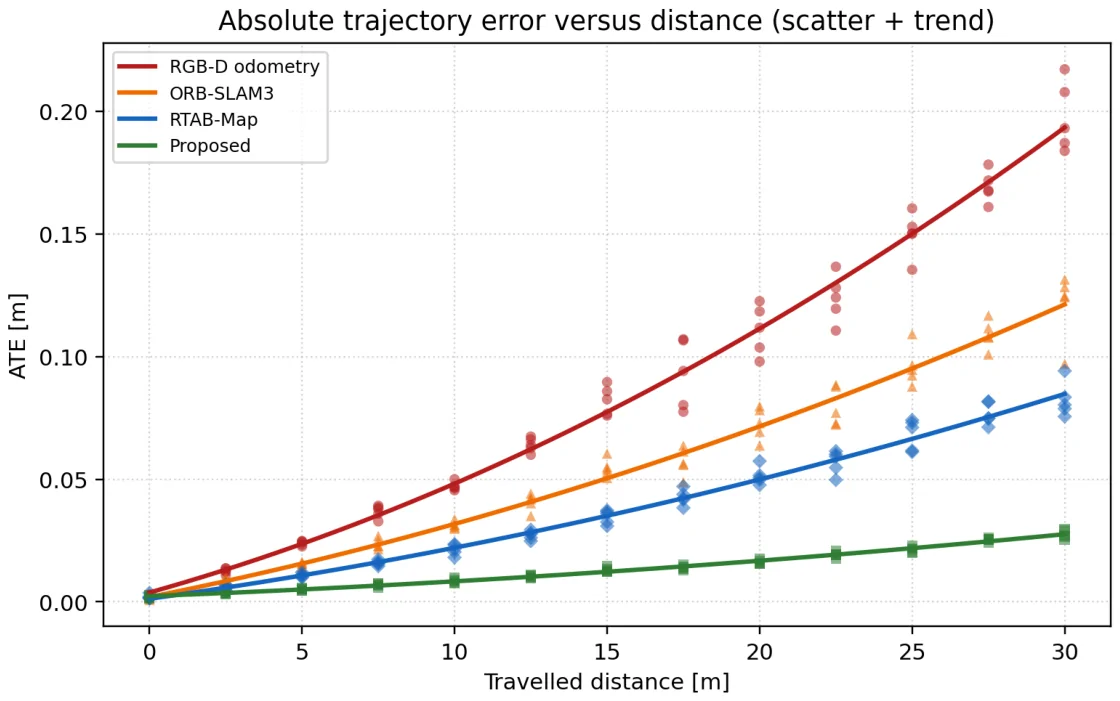
ATE growth versus traveled distance on long sequences.

Figure 11 shows ATE growth versus traveled distance on long sequences.

Baseline ATE grows nearly linearly, while the SC-PipeJE curve stays flatter because axial slip is corrected when structure re-enters the window rather than being integrated indefinitely. The distance trend matches the 67.6% ATE reduction versus RTAB-Map in Table 9 and confirms that continuous geometry plus landmarks improve long-horizon observability for Challenge 1. Paired tests over five repeats remain significant (p<0.01, 95% CI of the mean difference [0.068,0.076] m). The statistical unit and the meaning of the five repeats are defined in the Evaluation Protocol.

### 4.3. Damage Localization

If pose were perfect, single-frame mask back-projection would already be usable; it is not. Box-center projection ignores mask shape and mixes wall pixels with background depth, yielding 96.66 mm mean 3D error (Table 10). Replacing the box by a mask reduces the error to 61.53 mm, showing that segmentation quality helps, yet residual scatter remains large: depth holes at specular spots and a single poor pose sample can shift a damage by centimeters. A stronger non-joint baseline is fixed-pose multi-frame fusion (configuration C2): the same detections and associations are temporally fused while poses are held fixed, lowering 3D error to 38.4 mm. Full joint multi-frame estimation in SC-PipeJE further lowers the mean to 32.88 mm.

**Figure 12:**
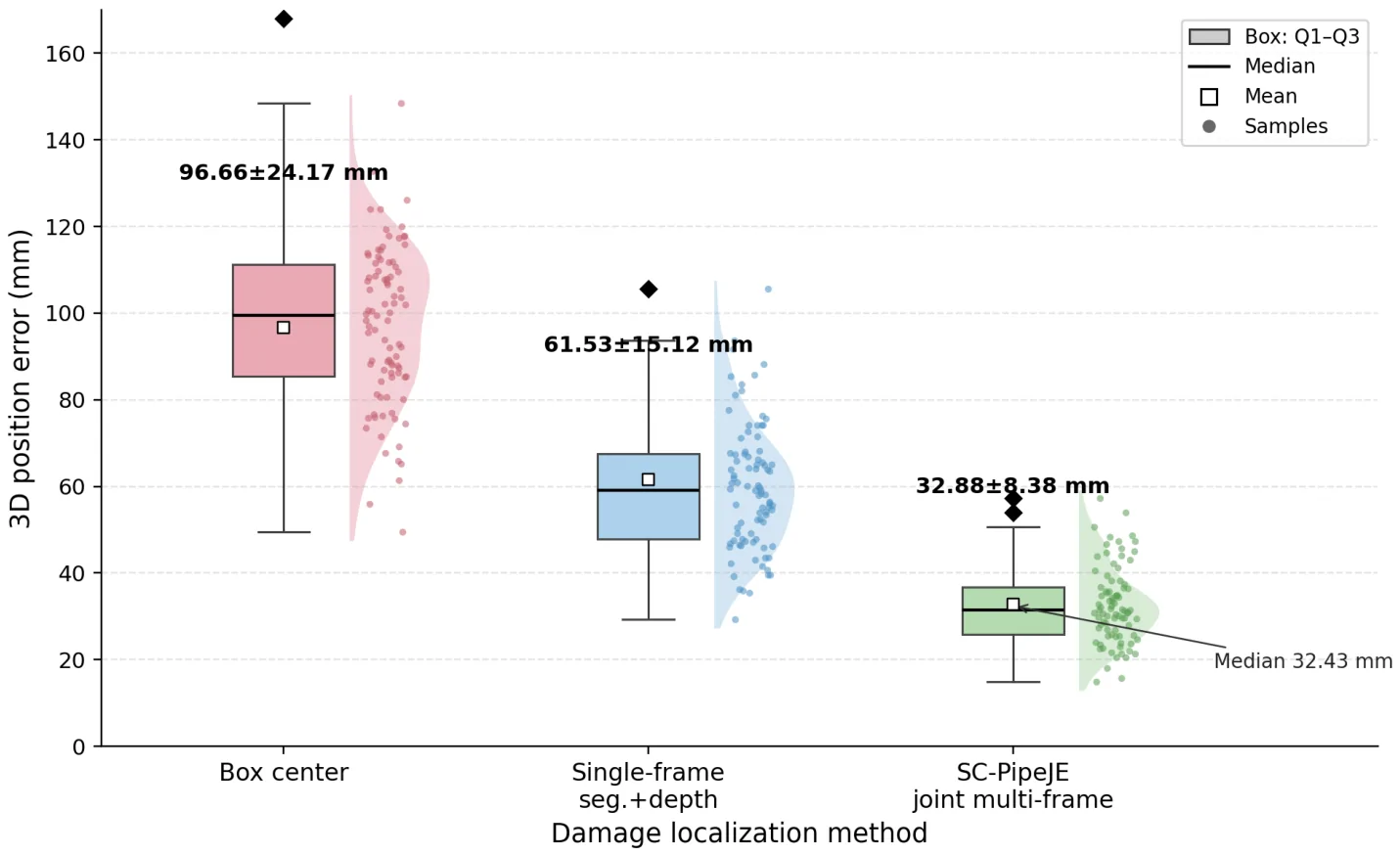
Error dispersion for 3D damage localization (English labels; box, whiskers and samples; means match Table 10).

Figure 12 shows the distributional effect for 3D damage error.

The interquartile range and whiskers shrink under multi-frame fusion, and cross-frame consistency of the same damage improves from 14.6 mm to 6.9 mm std. This behavior matches the 32.88 mm mean in Table 10 and shows that Challenge 2 is addressed by bidirectional pose–damage coupling rather than by passive back-projection. Axial and circumferential errors fall together (22.33 mm/2.69°). The paired comparison against single-frame back-projection is significant (p<0.01, 95% CI [24.1,33.2] mm).

**Figure 13:**
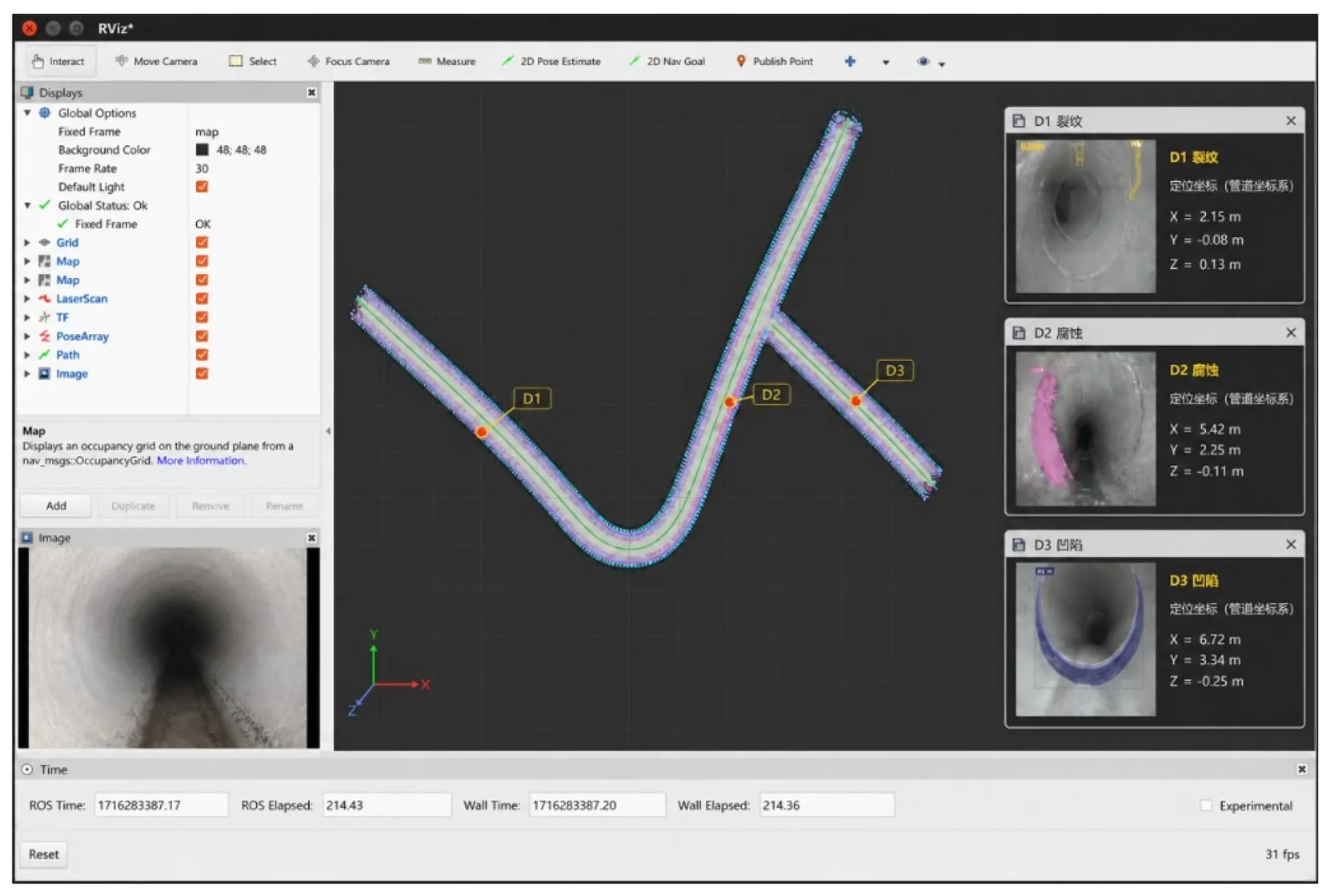
Qualitative pipe-frame localization interface: map with trajectory and damages $D_{1}$–$D_{3}$, live camera view, and pipe-coordinate readouts for crack, corrosion and dent.

Figure 13 shows the online inspection interface on a bend–tee segment: the reconstructed pipe-frame map with robot trajectory, live RGB view, and three localized damages (crack $D_{1}$, corrosion $D_{2}$ and dent $D_{3}$) reported with pipe-coordinate stations. The panel is consistent with the multi-frame gains in Table 10 and illustrates the maintenance-oriented output of SC-PipeJE beyond image-plane boxes.

### 4.4. Joint-Feedback Control and Extended Damage Baselines

To separate multi-frame fusion from bidirectional joint optimization, we compare three configurations under identical detection and association settings: (C1) structure-constrained pose estimation without damage factors; (C2) multi-frame damage fusion with poses held fixed (no damage residuals fed back into pose) and (C3) the complete joint optimization of SC-PipeJE. Table 11 reports ATE and 3D damage error. C1 matches ablation A3 (landmarks on, damage factors off). C2 keeps the C1 poses frozen and only fuses multi-frame damage states, so its ATE cannot improve over C1 by construction; the damage error drop from 46.1 to 38.4 mm isolates the gain of temporal fusion under fixed pose. C3 then re-enables damage→pose feedback and recovers both metrics (0.0343 m/32.88 mm), showing that the remaining ATE gain is not explained by fusion alone. On the test split, each retained damage is observed in 11.2±3.4 keyframes on average (median 10); damages seen in fewer than three frames contribute little axial anchoring. Injecting controlled cross-frame association swaps (Table 16) raises 3D damage error faster than ATE, consistent with incorrect tracks pulling $d_{m}$ while robust landmark factors still stabilize most of the trajectory. We further include a tracking-/fusion-style damage baseline beyond box-center and single-frame back-projection, and analyze how the number and axial distribution of damage observations affect ATE.

The ATE drop from 0.0770 m (C1/A3) to 0.0343 m (C3/A4) has a geometric reason, not only an empirical one. An ideal cylinder leaves axial translation free, so continuous geometry mainly stabilizes radial pose and scale. Discrete welds, elbows, and tees break that axial symmetry and bring ATE down to the C1 level (0.0770 m), but they remain sparse: between landmarks, the window can still accumulate slip along the centerline. Multi-frame damages that persist across keyframes act as *temporary axial anchors*: once a damage state $d_{m}$ is tied to several poses through depth-weighted residuals, correcting $d_{m}$ also pulls the intervening poses, which is exactly the damage → pose feedback disabled in C2 and enabled in C3. That is why C2 can improve 3D damage error (46.1 → 38.4 mm) while leaving ATE unchanged at 0.0770 m, whereas C3 halves ATE to 0.0343 m—fusion alone cleans the damage estimate; feedback is what returns axial constraint to the trajectory. Landmarks and damages are complementary rather than redundant: landmarks provide strong but infrequent resets; damages densify axial observability between resets when they are re-observed. Table 12 supports this reading. With landmarks held fixed, ATE falls as more multi-frame damages enter the window and as their mean axial spacing shrinks (e.g., 4 damages/≈5.2 m → 0.0614 m; full set/≈1.6 m → 0.0343 m). Sparse damages (≤4 per sequence, spacing >4 m) leave long unconstrained spans, so most of the C1-level drift remains; denser, more evenly spaced damages shorten those spans and saturate toward the full C3 result. Damages seen in fewer than three keyframes contribute little anchoring, consistent with needing repeated observations before $d_{m}$ can reliably constrain pose.

### 4.5. Ablation of Structure Constraints

Table 13 shows which constraint improves which degree of freedom. From A0 to A1, cylinders mainly pin the robot against the wall: ATE drops only from 0.219 to 0.193 m because an ideal cylinder remains free along its axis. Adding axis and radius (A2) tightens scale and heading enough to reach 0.139 m. The decisive step is A3: welds, elbows and tees break axial symmetry, and ATE falls to 0.077 m. Finally, A4 shows that multi-frame damages are not cosmetic—ATE halves again to 0.0343 m while 3D damage error moves from 46.1 to 32.88 mm. The FPS cost of the full stack is about 9 frames relative to A0, remaining near 20 FPS. Without discrete landmarks, a long straight pipe remains only partially observable, which is tested next.

### 4.6. Robustness Across Conditions

Stratified results in Table 14 show gradual rather than brittle degradation. Strong light is the easiest regime (ATE 0.029 m, damage 28.4 mm). Weak light first hurts detection (mAP@0.5:0.95 from 0.741 to 0.693), after which pose and damage follow because fewer reliable landmarks and masks enter the optimizer. Reflection/dirt and occlusion are more severe: depth holes corrupt cylinder fits, so ATE rises to 0.046 to 0.051 m and damage error to about 40 to 43 mm. Elbow/tee segments are geometrically harder than straight pipes but easier than heavy occlusion, because topology itself supplies landmarks. Across all listed conditions, ATE stays under 0.052 m, which remains below RTAB-Map’s overall 0.106 m on the main benchmark.

### 4.7. Landmark-Free Straight Pipes

**Figure 14:**
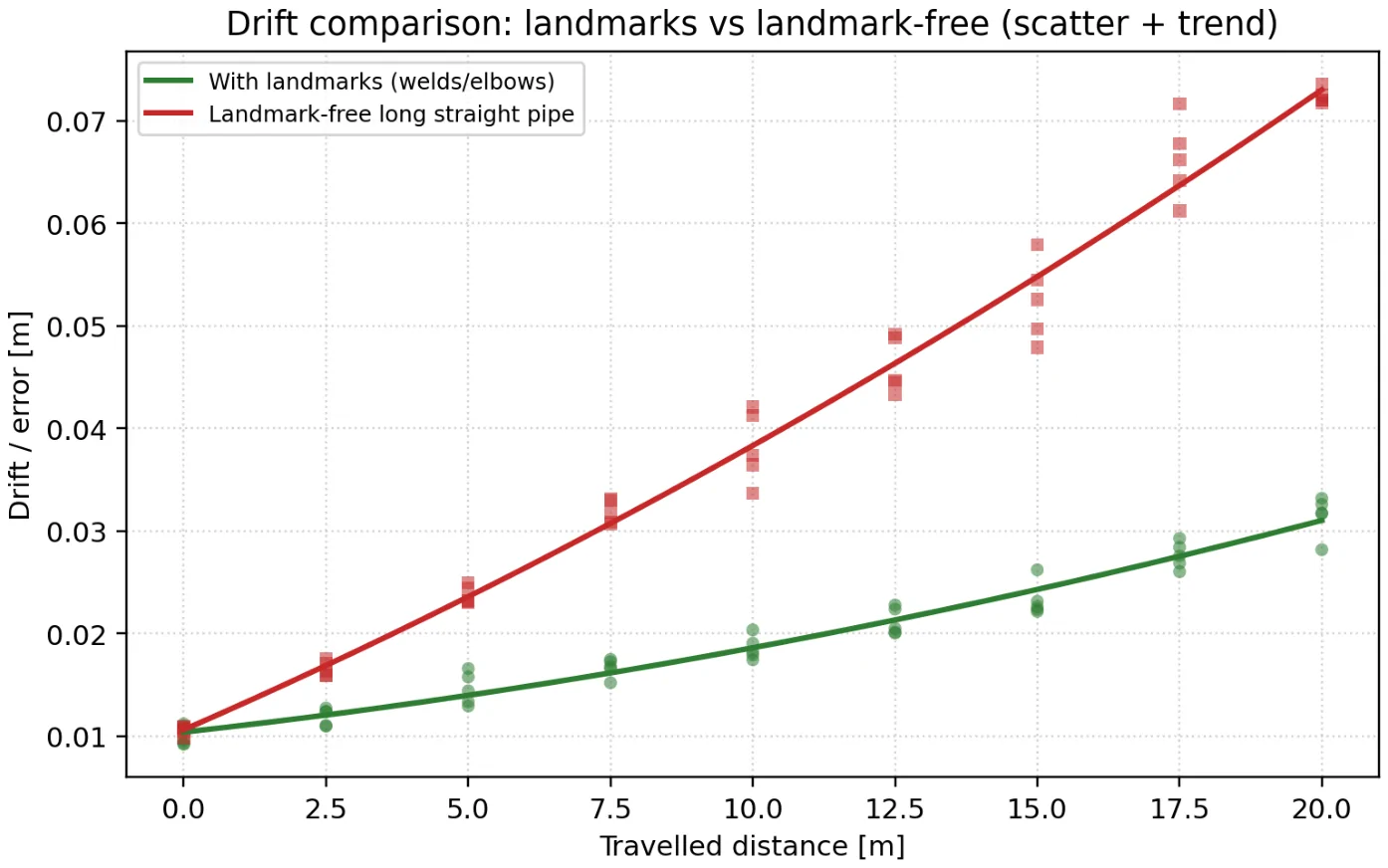
Axial drift with and without discrete structural landmarks.

Figure 14 compares axial drift with and without discrete structural landmarks on a long straight run.

When welds and elbows are removed, axial drift accelerates even with cylinder constraints active. Radial residuals cannot determine travel along a uniform tube, so the curve separation is an observability boundary rather than an implementation failure. The pattern is consistent with the A3 → A4 jump in Table 13 and shows why Challenge 1 requires discrete landmarks or inertial aiding on featureless spans.

### 4.8. Sensitivity, Operating Envelope and Extended Robustness

Table 15 sweeps the damage weight $\lambda_{d}$, the landmark weight $\lambda_{l}$ and the window size *N* around the defaults in Table 4. Mild detuning of $\lambda_{d}$ mainly hurts damage error; under-weighting $\lambda_{l}$ raises ATE because axial anchors weaken; enlarging *N* beyond 8 yields diminishing returns at higher compute. Table 16 then probes operating limits under controlled degradations on the same test sequences: depth dropout, landmark spacing/noise, mask occlusion, cross-frame association swaps, higher crawl speed and reduced landmark density. Accuracy degrades gracefully up to moderate stress; beyond roughly 50% depth dropout or 20% forced association error, both ATE and 3D damage error rise into a regime where IMU/wheel aiding would be advisable.

### 4.9. Sensitivity to Non-Circular Cross-Sections

Because the continuous factors fit a circular cylinder, we probe sensitivity to cross-section ovalization on the Corpus A test sequences. Wall points are anisotropically scaled in the pipe cross-section so that the major/minor axis ratio equals a/b, while landmarks, damages, depth timing and the estimator are otherwise unchanged; a/b=1 recovers the circular baseline. Table 17 reports ATE and 3D damage error.

The degradation is not uniform across degrees of freedom. Mild ovalization (a/b≈1.05) mainly inflates the cylinder residual and slightly biases the fitted radius/centerline, so ATE and damage error rise modestly. Moderate ovalization (a/b≈1.10–1.15) couples a wrong radial model into every window: radial pose and scale drift, and pipe-frame damage coordinates that depend on the fitted centerline inherit that bias, so 3D damage error grows faster than ATE. Strong ovalization (a/b≥1.20) makes the circular residual systematically inconsistent with the cloud; landmarks and multi-frame damages can still provide intermittent axial resets, yet they cannot correct a persistently wrong cross-section model, and both metrics leave the operating range of the circular Corpus A baseline. Thick deposits would act similarly by breaking local circularity. These probes, therefore, quantify the sensitivity requested by the reviewer and motivate deformable or piecewise cross-section models in future work.

### 4.10. Runtime and Complexity

End-to-end mean latency is 50.5 ms (≈19.8 FPS) on an RTX 3060 workstation (Table 18). Detection accounts for 28.4 ms; geometry and window optimization together cost about 21.7 ms. P95 remains under 59 ms, so occasional harder frames do not collapse real-time behavior at moderate crawl speeds. Further onboard acceleration should, therefore, target the segmenter, consistent with Equation (18).

### 4.11. Failure Cases

**Figure 15:**
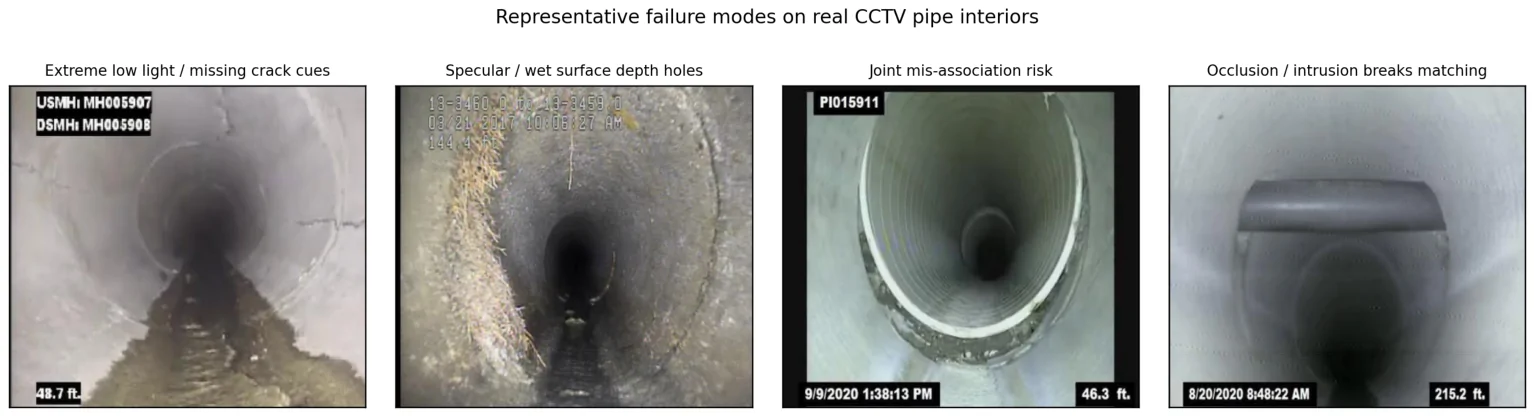
Representative failure cases on real closed-pipe CCTV frames.

Figure 15 shows representative remaining failures.

Extremely low light can still erase hairline cracks before enhancement helps; strong specular returns create depth holes that destabilize local cylinder fits; rare weld mis-associations inject a wrong axial anchor, and incorrect damage matching across frames can temporarily pull both $d_{m}$ and nearby poses. These modes justify the Huber kernels already used and motivate adaptive depth uncertainty and stricter landmark gating, without contradicting the average gains in Table 9 and Table 10.

### 4.12. On-Site RGB-D Qualitative Check

**Figure 16:**
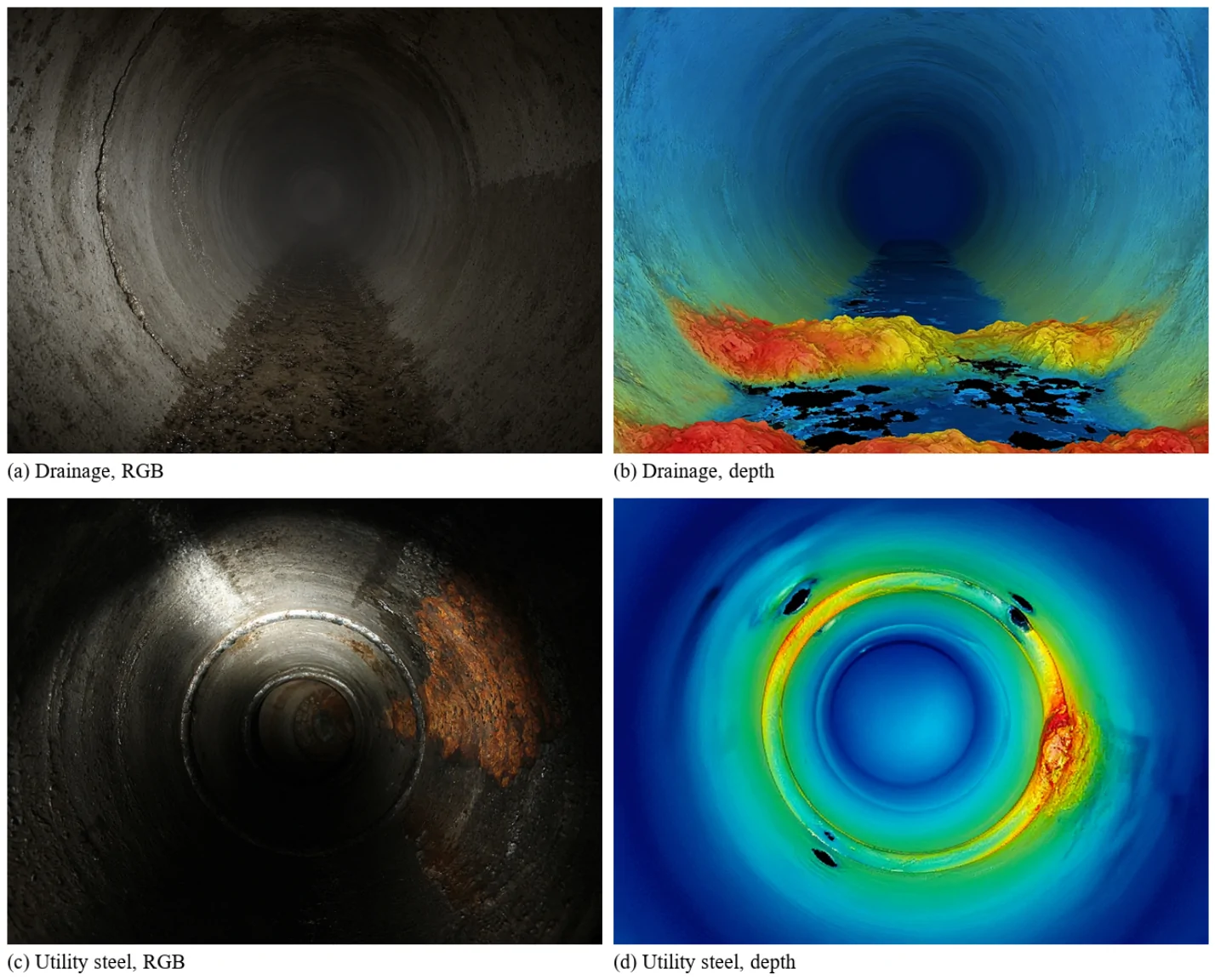
On-site RGB-D samples (qualitative). (**a**,**b**) buried drainage; (**c**,**d**) steel utility duct. No pipe-frame ground truth.

Corpus A is a laboratory 400 mm PVC rig, and Corpus B has no depth. We, therefore, recorded a small on-site RGB-D set with the same RealSense and crawler: two sites (buried concrete drainage; steel utility duct), three short runs, 186 frames, about 11.4 m at 0.06 m/s. No motion-capture or arm measurement was available, so this set is qualitative only and is not entered in Table 9 or Table 10. Figure 16 shows paired RGB and depth. Wet invert and specular steel produce more depth holes than the PVC rig; the cylinder fit still constrains the radial pose, while cracks, corrosion and weld rings remain visible to the detector. Axial error is not scored here.

## 5. Discussion

Three conclusions follow from the experiments. First, weakly textured pipes are not solved by stronger generic SLAM alone: appearance residuals lack axial observability, which is why ORB-SLAM3 and RTAB-Map improve over odometry yet still lag SC-PipeJE under the same RGB-D protocol. Second, continuous geometry and discrete landmarks play different roles—the former stabilizes radial pose, the latter resets axial drift—so omitting either changes the error mode, not merely its magnitude; this agrees with the geometric premise of CRORB and related cylinder/junction estimators [11,12,13], while our joint damage factors go beyond pose-only structure SLAM. Third, multi-frame damage coupling is bidirectional: better poses improve damage coordinates, and stable damages further regularize poses, which sequential detect-then-project pipelines cannot exploit.

The study also has clear boundaries. Same-protocol quantitative pose tables include generic RGB-D SLAM and the Cyl-RGB-D ablation. CRORB-RGBD is the same-protocol cylindrical-regularity reference (ATE 0.0814 m); it is not a copy of the published monocular scores, and Cyl-RGB-D is still only an internal ablation. Corpus A remains the only set with pipe-frame ground truth. The on-site RGB-D check (Section 4.12, 186 frames) shows drainage and steel appearance with depth, but without trajectory or damage ground truth, so it does not extend the quantitative claims. Laboratory circular sections further understate deformed, ovalized, or heavily deposited industrial pipes; Table 17 quantifies ATE/damage sensitivity under controlled ovalization and shows faster damage-error growth once the circular residual becomes inconsistent. Landmark-free straights remain only weakly observable without inertial aiding, and detection still dominates runtime on embedded hardware. Those limits define the next engineering steps: richer same-sensor structure-SLAM baselines, real-network validation, IMU/encoder fusion on long straights, non-circular pipe models and segmenter compression for board deployment.

## 6. Conclusions

### 6.1. Summary

This paper presented SC-PipeJE, a structure-constrained RGB-D estimator that jointly recovers in-pipe robot poses and pipe-frame 3D damage locations under weak texture in closed pipelines. By coupling continuous cylinder geometry, discrete structural landmarks and multi-frame damage factors in one sliding window, SC-PipeJE addresses axial observability and damage measurability as a single estimation problem rather than as cascaded post-processing. Experiments on Corpus A show competitive detection accuracy, substantially lower ATE than matched RGB-D SLAM baselines, and markedly improved 3D damage localization over single-frame back-projection at near real-time rates.

### 6.2. Limitations

The modeling assumptions remain restrictive. The continuous geometric factors presume locally circular cross-sections, so severe ovalization, collapse, or thick deposits violate the cylinder residual (Section 4.9). Discrete landmarks are treated as sparse axial anchors; long landmark-free straights, therefore, stay only partially observable from RGB-D alone. Multi-frame damage association further assumes stable instance identity across keyframes, which can break under intermittent occlusion or specular depth holes and temporarily bias both $d_{m}$ and nearby poses. CRORB-RGBD covers cylinder regularity on Corpus A but not an official CRORB binary, and the 186-frame on-site set has no ground truth, so neither replaces a larger field trial.

### 6.3. Future Work

Future work will follow these limitations directly. First, deformable or piecewise pipe models should replace the ideal cylinder on non-circular sections. Second, IMU or wheel-encoder factors should be fused on long landmark-free spans to restore axial observability without waiting for welds or elbows. The CRORB-style RGB-D port is limited to the six Corpus A test sequences; a longer comparison, and scoring on the on-site runs once ground truth exists, remain open. Adaptive depth uncertainty and stricter landmark gating should still harden association under specular returns. Segmenter compression for embedded deployment completes the roadmap.

## Figures and Tables

**Table 1:** Conceptual comparison of representative approaches (Y = supported; N = not in scope).

Approach	Modality	Struct. Constr.	Landmarks	Joint Pose–Dmg	Multi-Frame Dmg	Dmg → Pose
Generic RGB-D SLAM	RGB-D	N	N	N	N	N
CRORB-style pipe SLAM	Mono/vis.	Cylinder	Limited	N	N	N
Detect-then-project	RGB(+D)	N	N	N	Rare	N
**SC-PipeJE**	RGB-D	Cylinder + axis/rad.	Weld/elbow/tee	Y	Y	Y

**Table 2:** Corpus A statistics.

Item	Value
Total RGB-D frames	5468
Sequences (train/val/test)	18/4/6
Frames (train/val/test)	3827/821/820
Pipe inner diameter/material	400 mm/PVC
Total travel distance/nominal speed	47.6 m/0.08 m s^−1^
Landmarks (weld/elbow/tee)	42/18/12
Damages (crack/corrosion/dent/weld defect)	156/98/64/47

**Table 3:** Notation used throughout the paper (aligned with the original method formulation).

Symbol	Meaning
$T_{k}=\left\{R_{k},t_{k}\right\}$	Keyframe pose in the pipe frame
$S_{j}=\left\{c_{j},a_{j},r_{j}\right\}$/$M_{k}=\left\{c_{k},a_{k},r_{k}\right\}$	Local pipe/cylinder model
$L_{h}$, $d_{m}$	Structural landmark and damage position
$e_{t,k}$, $e_{R,k}$	Odometry translation and rotation residuals
$e_{c,ki}$, $e_{a,k}$, $e_{p,k}$, $e_{r,k}$	Cylinder, axis-direction, axis-position and radius residuals
$e_{l,kh}$, $e_{d,km}$	Landmark and multi-frame damage residuals
$w_{kmn}$	Depth uncertainty weight of mask pixels
s,θ,Δr	Axial, circumferential and radial damage parameters

**Table 4:** Optimization and association hyperparameters.

Parameter	Value
$\lambda_{t},\;\lambda_{R},\;\lambda_{c},\;\lambda_{a},\;\lambda_{p},\;\lambda_{r},\;\lambda_{l},\;\lambda_{d}$	1.0,1.0,0.5,0.8,0.8,0.5,2.0,1.5
Huber thresholds (odo/cylinder/landmark/damage)	0.10/0.05/0.08/0.10 m
Sliding-window size *N*/keyframe policy	N=8; keyframe if ∥Δt∥>0.05 m or ∥ΔR∥>5°
Cylinder points *P*/sampling	P=2000 uniformly subsampled wall points
LM termination (max iter/gradient tol.)	20 iterations/$10^{-6}$
Landmark/damage association thresholds	0.15 m/0.20 m (3D gating); mask IoU ≥0.3

**Table 5:** Training and inference flow of SC-PipeJE.

Stage	Procedure
Input	Sequence-split RGB-D frames, masks and intrinsics/extrinsics
Train detector	Optimize Equation (6) with AdamW; select checkpoint on validation mAP
Geometry	Back-project Equation (7); fit Equation (10); extract $L_{h}$
Optimize	Minimize Equation (16) over $\left\{R_{k},t_{k},L_{h},d_{m}\right\}$; report $D_{m}$

**Table 6:** Sequence-level ATE, RPE and 3D damage error of full SC-PipeJE on the six Corpus A test sequences (mean matches Tables 9 and 10).

Test Seq.	ATE [m]	RPE [m]	3D Dmg [mm]
S1	0.0281	0.000258	28.4
S2	0.0312	0.000271	30.1
S3	0.0354	0.000285	33.5
S4	0.0382	0.000296	35.2
S5	0.0408	0.000308	36.8
S6	0.0321	0.000280	33.3
Mean	0.0343	0.000283	32.88

**Table 7:** Comparison with YOLO-series and non-YOLO instance segmentation baselines.

Method	Family	Prec.	Rec.	mAP_50_	mAP_50:95_	Mask	FPS
YOLACT	Non-YOLO	0.8124	0.7710	0.8412	0.5521	0.5310	32.1
Mask R-CNN	Non-YOLO	0.8382	0.7964	0.8670	0.5830	0.5620	11.4
Cascade Mask R-CNN	Non-YOLO	0.8511	0.8082	0.8794	0.6012	0.5810	8.7
SOLOv2	Non-YOLO	0.8470	0.8021	0.8743	0.5924	0.5731	14.2
CondInst	Non-YOLO	0.8445	0.7990	0.8711	0.5880	0.5682	16.8
YOLOv5s-seg	YOLO	0.8413	0.7982	0.8691	0.5860	0.5652	34.2
YOLOv8-seg	YOLO	0.8564	0.8161	0.8831	0.6145	0.5947	28.4
YOLOv9-seg	YOLO	0.8682	0.8290	0.8964	0.6310	0.6102	26.5
YOLOv10-seg	YOLO	0.8721	0.8342	0.9010	0.6381	0.6180	29.1
YOLOv11n-seg	YOLO	0.8790	0.8413	0.9082	0.6470	0.6270	31.8
**SC-PipeJE**	YOLO-based	0.9177	0.8921	0.9415	0.7194	0.6980	20.5

**Table 8:** Detection and instance segmentation under progressive configurations (mean ± std).

Model	Prec.	Rec.	mAP_50_	mAP_50:95_	Mask	FPS
YOLOv8-seg	0.8564±0.0054	0.8161±0.0031	0.8831±0.0048	0.6145±0.0039	0.5947±0.0049	28.43±0.38
+Low-light/Attn	0.8820±0.0065	0.8419±0.0032	0.9009±0.0063	0.6420±0.0054	0.6188±0.0013	25.79±0.10
+BiFPN	0.8917±0.0053	0.8578±0.0029	0.9171±0.0074	0.6643±0.0049	0.6464±0.0057	23.91±0.25
+P2 head	0.9087±0.0038	0.8754±0.0081	0.9310±0.0016	0.6887±0.0028	0.6747±0.0028	21.68±0.32
**Full SC-PipeJE**	0.9177±0.0048	0.8921±0.0016	0.9415±0.0033	0.7194±0.0025	0.6980±0.0048	20.46±0.36

**Table 9:** Pose estimation results (Cyl-RGB-D ablation and same-protocol CRORB-RGBD).

Method	ATE [m]	RPE [m]	Drift Rate [%]
RGB-D odometry	0.2190±0.0124	0.002317±0.000049	1.4465
ORB-SLAM3	0.1461±0.0113	0.000862±0.000016	0.9648
RTAB-Map	0.1061±0.0013	0.000669±0.000010	0.7009
Cyl-RGB-D (struct. only)	0.1390±0.0086	0.000812±0.000021	0.918
CRORB-RGBD (cylinder regularity)	0.0814±0.0051	0.000548±0.000013	0.5377
**SC-PipeJE**	0.0343±0.0018	0.000283±0.000009	0.2268

**Table 10:** Damage localization accuracy (mean ± std; fixed-pose multi-frame fusion added).

Method	Axial [mm]	Circumferential [°]	3D [mm]
Box center	68.93±20.20	8.37±2.50	96.66±24.17
Single-frame seg.+depth	42.77±11.87	5.48±1.62	61.53±15.12
Fixed-pose multi-frame fusion (C2)	26.10±7.80	3.15±0.85	38.40±9.60
**SC-PipeJE joint multi-frame (C3)**	22.33±6.35	2.69±0.70	32.88±8.38

**Table 11:** Control experiment separating fixed-pose multi-frame fusion from joint feedback.

Configuration	ATE [m]	3D Dmg [mm]
C1: structure pose, no damage factors	0.0770	46.1
C2: fixed-pose multi-frame fusion	0.0770	38.4
C3: full joint SC-PipeJE	0.0343	32.88

**Table 12:** ATE versus number and mean axial spacing of multi-frame damage observations (landmarks fixed; test sequences).

Damage Setting	ATE [m]	3D Dmg [mm]
0 damages (C1)	0.0770	46.1
4 damages/mean spacing ≈5.2 m	0.0614	42.8
8 damages/mean spacing ≈3.1 m	0.0487	38.9
12 damages/mean spacing ≈2.0 m	0.0396	35.2
Full set (C3; 28 instances, mean spacing ≈1.6 m)	0.0343	32.88

**Table 13:** Ablation of geometric constraints and multi-frame damage factors.

Cfg	Cyl.	Axis/Rad.	Landmarks	Multi-Frame	ATE [m]	3D Dmg [mm]	FPS
A0	–	–	–	–	0.2190	91.8	29.0
A1	✓	–	–	–	0.1930	77.4	25.7
A2	✓	✓	–	–	0.1390	65.2	23.4
A3	✓	✓	✓	–	0.0770	46.1	20.9
A4	✓	✓	✓	✓	0.0343	32.88	19.8

**Table 14:** Robustness under illumination, disturbance and topology conditions.

Condition	Seq.	mAP_50:95_	Mask AP	ATE [m]	3D Dmg [mm]
Strong light	6	0.741	0.716	0.029	28.4
Weak light	6	0.693	0.671	0.038	34.9
Reflection/dirt	5	0.675	0.652	0.046	39.6
Occlusion	5	0.661	0.638	0.051	43.2
Elbow & tee	7	0.702	0.679	0.041	36.1

**Table 15:** Sensitivity of ATE and 3D damage error to $\lambda_{d}$, $\lambda_{l}$ and window size *N* (defaults in bold).

Setting	ATE [m]	3D Dmg [mm]
$\lambda_{d}\;=\;0.5$	0.0412	36.1
**$\lambda_{d}\;=\;1.5$ (default)**	**0.0343**	**32.88**
$\lambda_{d}\;=\;3.0$	0.0368	31.4
$\lambda_{l}\;=\;1.0$	0.0485	35.2
**$\lambda_{l}\;=\;2.0$ (default)**	**0.0343**	**32.88**
$\lambda_{l}\;=\;4.0$	0.0331	33.6
N=4	0.0427	37.5
**N=8 (default)**	**0.0343**	**32.88**
N=12	0.0338	32.1

**Table 16:** Quantitative operating-envelope probes (controlled degradations on Corpus A test sequences).

Degradation	ATE [m]	3D Dmg [mm]
Baseline (full SC-PipeJE)	0.0343	32.88
Depth dropout 15%	0.0391	36.4
Depth dropout 30%	0.0476	41.2
Depth dropout 50%	0.0684	52.7
Landmark spacing ×2/detection noise +5 cm	0.0589	39.6
Landmark spacing ×3	0.0712	43.5
Mask occlusion (area −40%)	0.0458	48.2
Cross-frame association error 5%	0.0379	38.6
Cross-frame association error 10%	0.0412	44.8
Cross-frame association error 20%	0.0556	58.3
Crawl speed ×1.5 (0.12 m s^−1^)	0.0402	35.8
Landmark density −50%	0.0623	41.0

**Table 17:** Sensitivity to cross-section ovalization (major/minor axis ratio a/b; controlled warp of Corpus A test clouds).

Cross-Section	ATE [m]	3D Dmg [mm]
Circular baseline (a/b=1)	0.0343	32.88
Mild ovalization (a/b=1.05)	0.0398	36.5
Moderate ovalization (a/b=1.10)	0.0486	44.2
Stronger ovalization (a/b=1.15)	0.0612	53.7
Severe ovalization (a/b=1.20)	0.0795	67.4

**Table 18:** Runtime breakdown.

Module	Params [M]	FLOPs [G]	Mean [ms]	P95 [ms]
Detection & segmentation	8.7	24.6	28.4	33.8
Point-cloud geometry	–	–	9.6	12.4
Sliding-window optimization	–	–	12.1	16.7
End-to-end	–	–	50.5	58.9

## Data Availability

The datasets generated during this study are available from the corresponding author upon reasonable request.
